# Tracking the impact of maternal health service coverage on maternal mortality across 137 low- and middle-income countries in the progress toward Sustainable Development Goals

**DOI:** 10.7189/jogh.16.04324

**Published:** 2026-10-02

**Authors:** Francis Sarial Ganancial, Vivian Chia-Rong Hsieh

**Affiliations:** 1 Department of Public Health, China Medical University, Taichung, Taiwan; 2 Department of Health Services Administration, China Medical University, Taichung, Taiwan

**Keywords:** developing countries, health services accessibility, maternal health, maternal health services, maternal mortality ratio

## Abstract

**Background:**

Achieving Sustainable Development Goal (SDG) 3, particularly targets 3.1 (reducing the global maternal mortality ratio (MMR) to less than 70 per 100,000 live births) and 3.8 (universal health coverage), requires sustained reductions in maternal mortality and equitable access to essential maternal health services (MHS). Despite global progress, large disparities persist. Low- and middle-income countries (LMICs) account for more than 90% of maternal deaths worldwide, largely reflecting gaps in access to essential MHS. However, multi-country evidence examining how improvements in MHS during the SDG period translate to reductions in MMR remains limited. This study assessed disparities in MHS coverage and their associations with MMR across LMICs.

**Methods:**

We conducted a longitudinal panel analysis of 137 LMICs, using country-level data from the World Health Organization Global Health Observatory and the World Bank from 2015 to 2023. Indicators included coverage of at least four antenatal care visits (ANC4+), facility-based delivery (FBD), and skilled birth attendance (SBA). We estimated associations between MHS coverage and MMR using generalised linear mixed models with a gamma-log link, with analyses stratified by country income group and maternal education.

**Results:**

The median MMR of the 137 LMICs declined from 109.3 to 85.5 deaths per 100,000 live births from 2015 to 2023. Coverage of FBD and SBA increased by 5.6% and 5.0%, respectively. Higher coverage of ANC4+ (*β* = −0.01, *P* = 0.02) and FBD (*β* = −0.01, *P* = 0.03) was significantly associated with lower MMR. These associations were stronger in lower-middle-income and upper-middle-income countries than in low-income countries. Stratified analyses showed larger reductions in MMR among women with moderate-to-high levels of maternal education.

**Conclusions:**

Although maternal mortality decreased during the SDG period, progress remains insufficient and inequitable. Strengthening equitable access to essential MHS is critical to accelerating MMR reductions and supporting progress toward SDG targets in LMICs.

Reducing maternal mortality and improving maternal health are core objectives of the Sustainable Development Goals (SDGs), specifically SDG 3, which commits countries to lowering the global maternal mortality ratio (MMR) to fewer than 70 per 100,000 live births by 2030 and ensuring that no country exceeds 140 per 100,000 live births (target 3.1). It also emphasises universal health coverage (UHC), including equitable access to essential maternal health services (MHS) (target 3.8) [1–4]. The SDGs are grounded in a commitment to reducing inequalities both within and between countries, particularly the persistent gap between high-income (HICs) and low- and middle-income countries (LMICs), guided by the principle of ‘leaving no one behind’ [1,5,6]. Maternal health during pregnancy and childbirth reflects the functioning of the broader health system, as preventing maternal deaths requires coordinated and interdependent components, including skilled health personnel, infrastructure, financing, and referral systems [2,4,7]. Achieving the 2030 targets ultimately depends on equitable and sustained access to effective MHS across the continuum of care, such as antenatal care (ANC), skilled birth attendance (SBA), institutional delivery (FBD), postnatal care (PNC), and family planning [2,4,8,9].

Despite decades of global efforts to improve maternal health, maternal mortality remains a leading cause of death among women of reproductive age [9–11]. An estimated 260,000 women died during or after pregnancy and childbirth in 2023, with 92% of these deaths occurring in LMICs [3,10–12]. Remarkable disparities persist, with MMRs nearly 35 times higher in LMICs than in HICs (346 *vs.* 10 deaths per 100,000 live births) [3]. HICs have achieved very high coverage of essential MHS, including SBA (99%) and ANC (96%), supported by well-resourced health systems that provide timely and appropriate obstetric care [13,14]. In contrast, coverage of these services remains substantially lower in many LMICs, particularly in sub-Saharan Africa and Southern Asia, where ANC coverage ranges from approximately 50% to 58% [15]. These regions also account for the majority of global maternal deaths [3,12,16,17], reflecting structural constraints in health system capacity and socioeconomic conditions that limit equitable access to care [11,17–20].

Many studies have also shown that higher MHS coverage and socioeconomic factors, including maternal education and health financing schemes, are strongly associated with reductions in MMR [11,21–25]. Although progress in MHS coverage has been seen in LMICs over the years [11,18,20,21], substantial inequalities persist both between and within countries, especially across income-level groups. However, the existing evidence remains insufficient to guide policy, as most studies rely on cross-sectional or single-country analyses. For example, studies conducted in the Philippines and India have reported that increased utilisation of MHS, including ANC, FBD, and PNC, is associated with reductions in maternal mortality within national contexts [21,22]. While informative, these studies are context-specific and limit generalisability. In contrast, large-scale studies often focus on descriptive trends or projections of MMR and maternal disease burden [12,16,17,26] with less emphasis on directly quantifying the relationship between MHS coverage and MMR. Furthermore, few longitudinal multi-country studies have examined how disparities in MHS coverage across income groups and maternal education are associated with maternal mortality during the SDG era. These evidence gaps limit understanding of whether improvements in MHS coverage translate into equitable reductions in MMR across LMICs. Therefore, this study aims to provide a longitudinal, multi-country analysis to examine disparities in MHS coverage and assess their association with MMR, thereby offering updated, policy-relevant evidence to inform maternal healthcare policies across LMICs.

## METHODS

In this study, we adhered to the Journal of Global Health’s GRABDROP guidelines [27] and followed the STROBE guidelines for reporting observational studies to ensure transparency and methodological rigour (Files S1 and S2 in the **Online Supplementary Document**) [28].

### Data sources, settings, and sample size

We conducted a secondary analysis using annual, country-aggregated data obtained from the World Health Organization Global Health Observatory (WHO GHO) and the World Bank from 2015 to 2023, which provide widely used health indicators. [29,30]. We obtained data on MHS indicators from the GHO [29], while we derived income classifications from the World Bank.

We treated each LMIC as a unit of analysis. We defined income groups according to the World Bank income classification based on gross national income (GNI) *per capita* using the Atlas method [30], and LMICs as countries with a GNI *per capita* below USD 12,735 in 2015. Within this group, low-income countries (LICs) had a GNI *per capita* of USD 1,045 or less; lower-middle-income countries (LMCs) had a GNI per capita between USD 1,046 and USD 4,125; and upper-middle-income countries (UMCs) had a GNI per capita between USD 4,126 and USD 12,735. Based on these criteria, we included 137 LMICs, comprising 31 LICs, 51 LMCs, and 55 UMCs.

### Study variables

#### Outcome variable

Our outcome variable was MMR, defined by WHO as the number of maternal deaths per 100,000 live births within a given period, depicting the risk of death from pregnancy-related causes, excluding accidental or incidental causes [10].

#### Explanatory variables

Our main explanatory variable was MHS coverage, and here it included at least four antenatal care visits (ANC4+), births attended by SBA, and institutional delivery. We excluded postnatal care due to substantial missing data (approximately 30%).

Based on WHO definitions, ANC coverage is the percentage of women aged 15-49 who received ANC4+ before a live birth. Adequate ANC supports routine pregnancy monitoring, early detection and management of pregnancy-related complications, and delivery of preventive interventions. SBA is the percentage of births attended by professional health personnel, including doctors, nurses, and midwives, who are equipped to manage deliveries and obstetric emergencies appropriately. FBD refers to the percentage of births delivered in healthcare facilities, where timely emergency obstetric care and effective referral systems are available. Additional covariates included densities of nurses, midwives, and physicians (per 10,000 population), out-of-pocket expenditure (percentage of current health expenditure), current health expenditure (CHE) (percentage of gross domestic product), and female literacy [30] (percentage of women ages 15 and above who can both read and write), used as a proxy for maternal education. We treated all covariates and explanatory variables as continuous.

### Statistical analysis

We conducted exploratory data analysis to assess missing values, outliers, and non-normality in the panel data. We imputed missing data using the most recent available value (last observation carried forward) to maximise data availability and preserve country-year observations [31]. The extent of missing data varied across study variables before imputation (Table S1 in the **Online Supplementary Document**). We assessed normality using the Kolmogorov-Smirnov test.

Due to the non-normality, we summarised descriptive statistics using the median (Mdn) and interquartile range (IQR). For multivariate analysis, we used generalised linear mixed models (GLIMMIX) with a gamma-log link function to assess associations between MHS coverage and MMR, adjusting for covariates. We selected the gamma-log specification because the outcome variable was a positive, right-skewed continuous variable. We informed model selection by residual diagnostics, model convergence, and fit statistics, including the −2log likelihood, Akaike and Bayesian information criterion. We tested interaction terms to assess whether the effect of ANC4+ varied by maternal education. We assessed multicollinearity among explanatory variables using variance inflation factors and tolerance statistics (Table S2 in the **Online Supplementary Document**). We used the country population size as a weight. Exponentiated regression coefficients (exp(*β*)) represent multiplicative effects on MMR and were converted to percentage change as (exp(*β*)−1) × 100%. Statistical significance was set at *P* < 0.05.

We conducted stratified analyses by maternal education level (low, moderate, high) across income groups to assess disparities. We performed sensitivity analysis using unweighted models to evaluate the robustness of the findings. We mapped MMR distribution using QGIS, version 3.34.0-Prizren (QGIS Development Team, Open-Source Geospatial Foundation Project, Chicago, Illinois, USA). We performed all analyses using SAS version 9.4 (SAS Institute Inc., Cary, North Carolina, USA) and *R*, version 4.4.2. (R Core Team, Vienna, Austria).

## RESULTS

### Sample characteristics and distribution

We included a total of 137 LMICs, of which 31 were LICs, 51 were LMCs, and 55 were UMCs, based on the 2015 World Bank income classification. Although we classified countries according to their 2015 income status, 28 countries (20.4%) shifted income classifications between 2015 and 2023 (Table S3 and S4 in the **Online Supplementary Document**).

### Trends in maternal mortality in LMICs

We observed trends in MMR across the 137 LMICs from 2015 to 2023 (Figure S1 in the **Online Supplementary Document**). Overall, the median MMR declined from 109.3 deaths per 100,000 live births in 2015 to 85.5 in 2023, representing an absolute reduction of 23.9 and a relative decrease of 21.8%, with an average annual percentage change (AAPC) of 3.0% (**Table 1**).

**Table 1:** Maternal mortality ratio per 100,000 live births of 137 low- and middle-income countries between 2015 and 2023 by World Bank income classification

	2015	2023			
**World Bank income classification**	**Mdn (IQR)**	**Mdn (IQR)**	**Absolute change**	**Crude % of change**	**AAPC**
Total	109.3 (41.5, 297.9)	85.5 (35.6, 225.0)	−23.9	−21.8	−3.0
Low	473.0 (353.8, 641.7)	353.6 (229.5, 505.4)	−119.4	−25.2	−3.6
Lower-middle	127.8 (51.9, 210.1)	93.7 (40.5, 155.0)	−34.1	−26.7	−3.8
Upper-middle	46.2 (21.4, 85.9)	42.1 (15.8, 74.7)	−4.1	−8.9	−1.2

AAPC – average annual percentage change, IQR – interquartile range, Mdn – median

Disaggregation by income group showed distinct patterns (Figure S2 in the **Online Supplementary Document**). We observed the largest absolute reduction among LICs, where the median MMR declined by 119.4 deaths per 100,000 live births, from 473.0 in 2015 to 353.6 in 2023. In contrast, LMCs experienced the greatest relative decline, at 26.7%, and the highest AAPC, at 3.8%, among the income groups (**Table 1**). Despite the overall downward trend, we observed a temporary increase in median MMR among LMCs and UMCs during the COVID-19 pandemic in 2021, followed by a decline in subsequent years (Figure S2 in the **Online Supplementary Document**).

We also observed substantial variation at the country level. South Sudan had the largest absolute decline, from 1,436 deaths per 100,000 live births in 2015 to 691.8 in 2023, a reduction of 744.2 deaths per 100,000 live births. By contrast, Belarus showed the smallest absolute change, declining slightly from 1.5 to 1.1 deaths per 100,000 live births and consistently the lowest MMR among LMICs. In relative terms, Eswatini recorded the largest percentage reduction, with a 58.3% decrease and an AAPC of 10.4%. Conversely, Venezuela, despite being classified as UMC, experienced the largest increase in MMR, rising from 136.3 to 226.7 deaths per 100,000 live births (Table S5 in the **Online Supplementary Document**).

High MMR levels were concentrated in many countries in sub-Saharan Africa, with most countries exceeding 220 deaths per 100,000 live births in 2015. By 2023, several countries in Southern and Southeast Asia, as well as parts of Latin America, shifted toward lower MMR categories, indicating improvements in maternal mortality. However, persistently high MMR levels remained concentrated in parts of sub-Saharan Africa (**Figure 1**).

**Figure 1:**
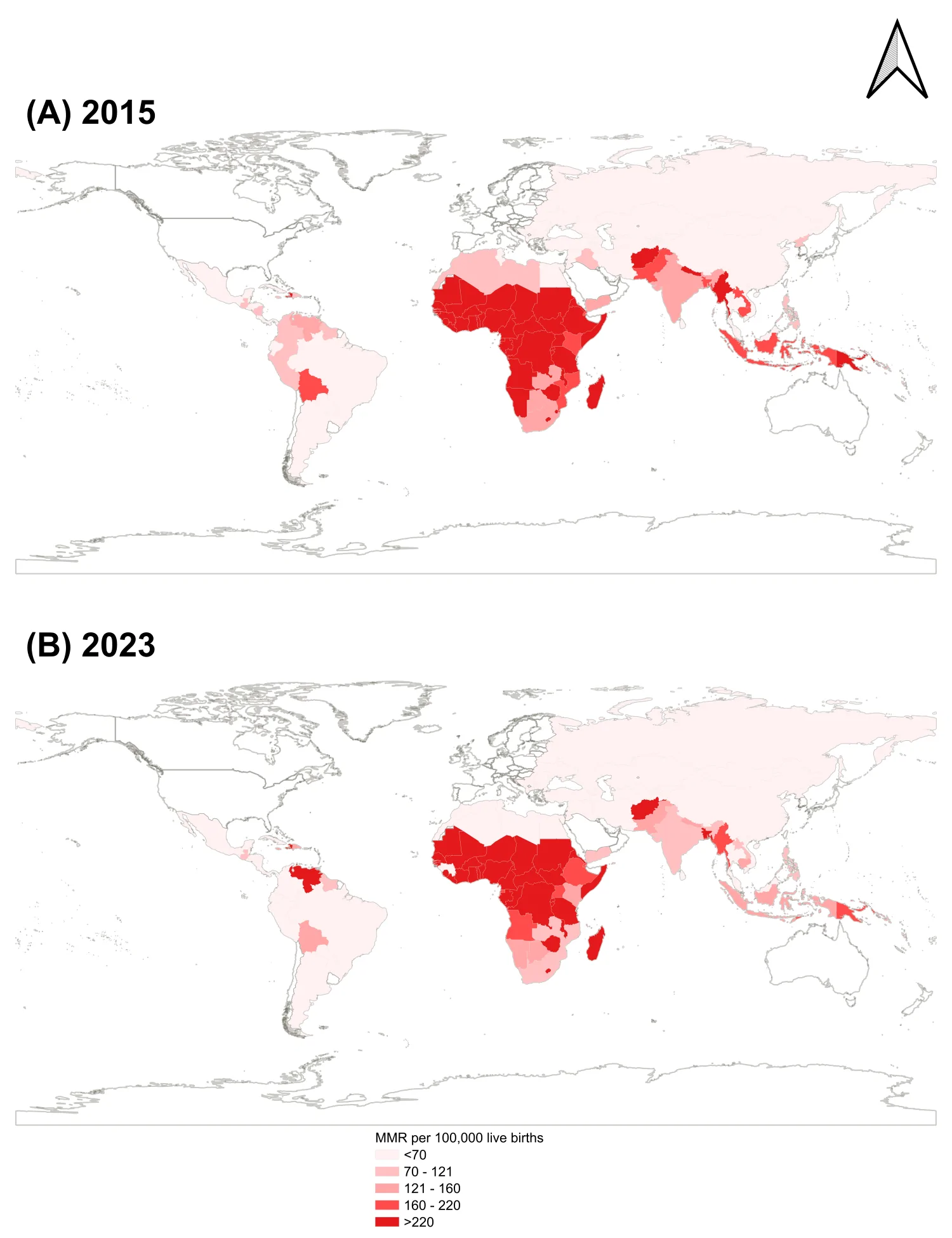
Geographical distribution of maternal mortality ratio in 137 low- and middle-income countries in 2015 and 2023. Generated using QGIS, version 3.34.0-Prizren.

### MHS coverage in LMICs

Coverage increased across all indicators, with the largest gains observed in SBA and FBD. SBA increased from a median of 91.0% (IQR = 68.0, 99.0) in 2015 to 96.0% (IQR = 83.0, 100) in 2023. Similarly, FBD increased from 87.4% (IQR = 63.0, 98.0) in 2015 to 93.0% (IQR = 80.1, 98.6). In contrast, ANC4+ coverage remained comparatively lower, increasing from a median of 75.0% (IQR = 55.1, 87.1) in 2015 to 77.8% (IQR = 61.3, 88.3) in 2023 (Figure S3 in the **Online Supplementary Document**).

When disaggregated by income group, we observed clear differences in coverage across indicators. For ANC4+, coverage increased across all income groups between 2015 and 2023, although LICs consistently had lower median coverage and greater variability than higher-income groups. LMCs demonstrated moderate improvements, while UMCs maintained the highest coverage levels in both years. For SBA, coverage improved across all income groups. LICs experienced notable increases between 2015 and 2023, whereas LMCs and UMCs maintained substantially higher coverage levels with relatively narrower distributions across countries. We observed a similar pattern for FBD. Coverage increased across income groups over time, particularly in LICs, although higher-income groups showed higher and more consistent levels (**Figure 2**). Substantial differences in MHS coverage across income groups persisted (Table S6 in the **Online Supplementary Document**).

**Figure 2:**
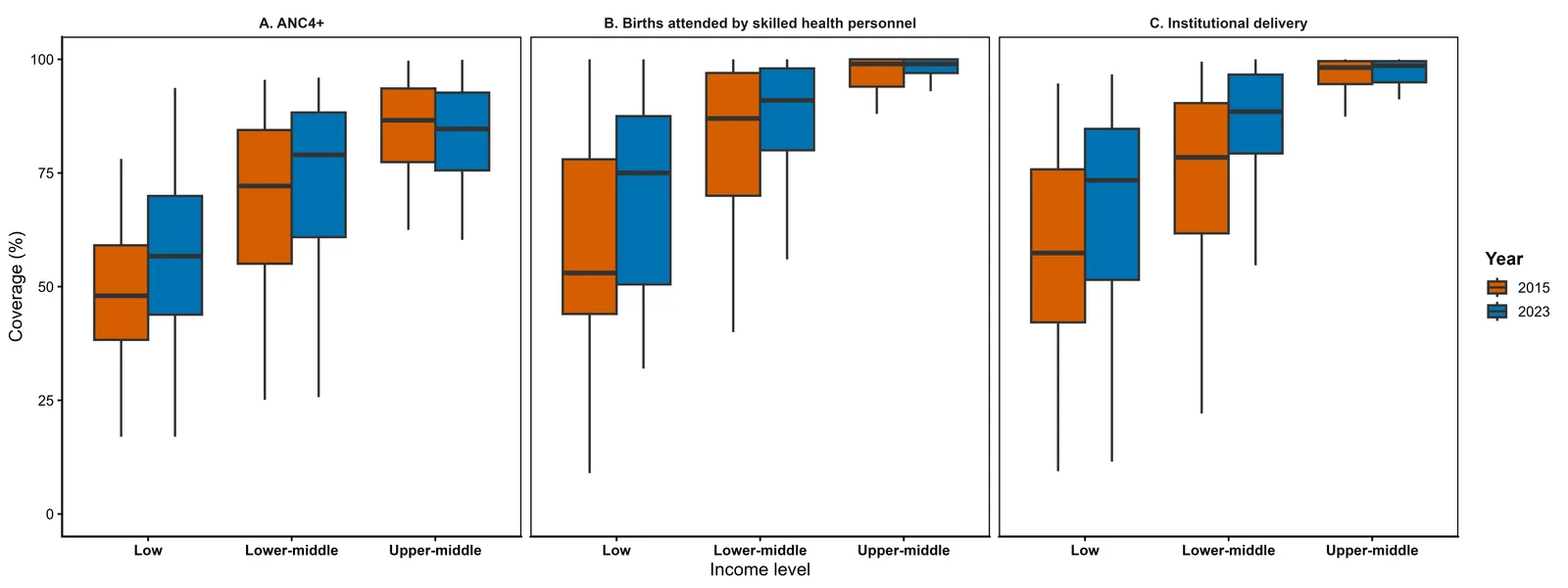
Distribution of maternal health services coverage in low- and middle-income countries between 2015 and 2023 by World Bank income group. ANC4+ – at least four antenatal visits.

Country-level distributions indicate higher coverage in 2023 (Figures S4–6 in the **Online Supplementary Document**). Delivery-related services, such as SBA and FBD, were consistently higher and showed lower variability than ANC4+ across all income groups. SBA and FBD achieved 99%–100% coverage in most UMCs, including Libya, China, and Turkmenistan (Tables S8–9 and Figures S5–6 in the **Online Supplementary Document**), whereas ANC4+ remains substantially lower and more dispersed in several countries, such as Cuba, Myanmar, and Mauritania (Table S7 and Figure S4 in the **Online Supplementary Document**). Although SBA and FBD coverage increased in LICs, from 53.0% to 75.0% in 2015, and from 57.4% to 73.4% in 2023, ANC4+ remained comparatively lower and showed marked variation in coverage across countries and income groups (Table S6 and Figures S4–6 in the **Online Supplementary Document**).

### Associations between MHS coverage and MMR in LMICs

We assessed the associations between MHS coverage and MMR using GLIMMIX models with a gamma-log link function, and expressed the model estimates as exponentiated coefficients (Figures S7–8 in the **Online Supplementary Document**).

In model 1, higher ANC4+ coverage (*β* = –0.01, *P* = 0.02) and FBD (*β* = –0.01, *P* = 0.03) were significantly associated with lower MMR. Among health system capacity indicators, densities of nurses and midwives (*β* = –0.01, *P* < 0.001) and physician density (*β* = −0.02, *P* = 0.01) were also significantly associated with lower MMR. Additionally, maternal education was significantly negatively associated with MMR (*β* = −0.01, *P* < 0.001). We incorporated an interaction term between maternal education and ANC4+ in model 2 to further examine whether the association between ANC4+ coverage and MMR varies by maternal education. In this model, FBD remained significantly associated with lower MMR (*β* = −0.01, *P* = 0.02), whereas the associations with ANC4+ (*β* = −0.01, *P* = 0.12) and maternal education (*β* = 0.004, *P* = 0.53) were no longer statistically significant. The interaction between maternal education and ANC4+ was statistically significant (*β* = −0.0002, *P* = 0.01) (**Table 2**). Sensitivity analyses using unweighted models yielded findings similar to those of the primary weighted analyses, although with some differences in the statistical significance of individual predictors (Table S10 in the **Online Supplementary Document**).

**Table 2:** Associations between maternal health-related indicators and maternal mortality ratio in low- and middle-income countries, 2015−2023*

	Model 1		Model 2	
	***β***	***P*-value**	***β***	***P*-value**
**Maternal health services**				
Antenatal care visits, at least four	−0.01	0.02	0.01	0.12
Births attended by skilled health personnel	−0.001	0.76	−0.002	0.49
Institutional delivery	−0.01	0.03	−0.01	0.02
**Health system capacity**				
Density of nurses and midwives	−0.01	<0.001	−0.01	<0.001
Density of physicians	−0.02	0.01	−0.01	0.03
Out-of-pocket expenditure	−0.003	0.30	−0.004	0.11
**Socioeconomic factors**				
Current health expenditure	0.03	0.07	0.02	0.22
Maternal education	−0.01	<0.001	0.004	0.53
**Interaction**				
Maternal education × antenatal care visits, at least four			−0.0002	0.01
**Income classification**				
Low income	ref		ref	
Lower-middle income	−0.52	0.001	−0.55	<0.001
Upper-middle income	−0.86	<0.001	−0.91	<0.001
**Year**				
2015	ref		ref	
2016	−0.01	0.24	−0.02	0.07
2017	−0.03	0.10	−0.04	0.04
2018	−0.02	0.35	−0.04	0.13
2019	−0.05	0.05	−0.07	0.02
2020	−0.02	0.79	−0.04	0.53
2021	0.31	<0.001	0.28	<0.001
2022	0.002	0.98	−0.01	0.86
2023	−0.13	0.002	−0.14	0.002
**Observations = 1,233**		1,067		1,067
**N = 137**		123		123

ref – reference

*Model 1 presents the fully adjusted generalised linear mixed model, including maternal health service indicators, health system capacity variables, socioeconomic factors, income classification, and year. Model 2 extends Model 1 by incorporating an interaction term between maternal education and the number of antenatal care visits (at least 4). We specified country-level random effects to account for within-country correlation over time.

### Stratified by income classification and maternal education level

ANC4+ coverage was negatively associated with MMR among women with high education across all income groups, with significant associations in LICs and UMCs (**Figure 3**). In LICs, higher ANC4+ coverage among women with high education was significantly associated with a lower MMR (*β* = −0.07, *P* = 0.03). In LMCs, FBD was significantly associated with a reduced MMR among women with moderate education (*β* = −0.01, *P* = 0.01). In UMCs, both ANC4+ coverage (*β* = −0.02, *P* < 0.001; *β* = −0.02, *P* = 0.001) and institutional delivery (*β* = −0.03, *P* < 0.001; *β* = −0.04, *P* = 0.02) were significantly associated with lower MMR among women with moderate-to-high education. Estimates for the low-education stratum in UMCs were unavailable because the model did not converge due to insufficient observations in this subgroup (Table S11 in the **Online Supplementary Document**).

**Figure 3:**
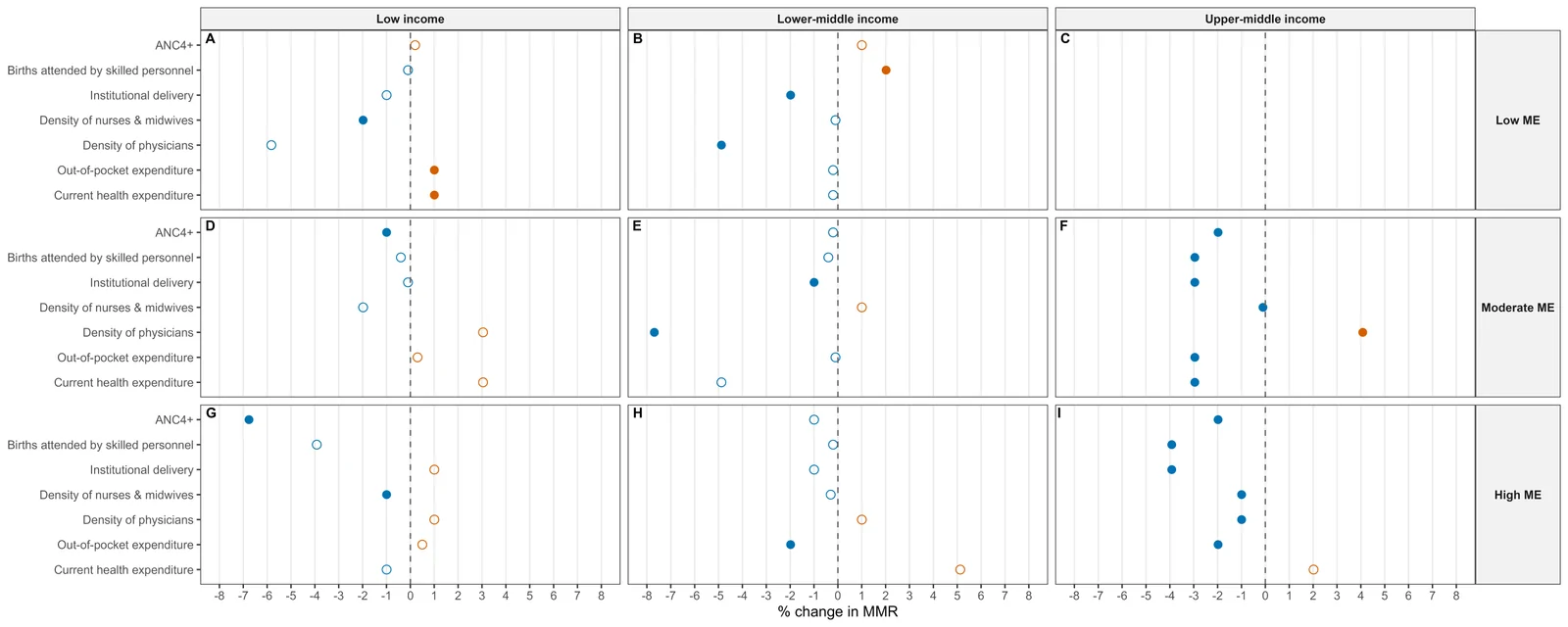
Multivariate-adjusted associations between maternal health services and maternal mortality ratio in low- and middle-income countries from 2015 to 2023, by income group and maternal education level. **Panels A–I** are arranged by income group from left to right (low, lower-middle, and upper-middle income) and by maternal education level from top to bottom (low, moderate, and high). Results for women with low ME in upper-middle-income countries are not presented because the corresponding stratified model failed to converge, likely due to limited data in this subgroup. Blue points indicate a decrease in maternal mortality ratio, whereas orange points indicate an increase. Filled points indicate statistical significance (*P* < 0.05), whereas open points indicate non-significance (*P* > 0.05). ANC4+ – at least four antenatal care visits, OOP – out-of-pocket, ME – maternal education.

## DISCUSSION

### Key findings

We examined disparities in MHS coverage and their associations with MMR across 137 LMICs between 2015 and 2023. Overall, maternal mortality declined, although progress varied across income groups and countries. Coverage of essential MHS also improved over time, particularly for SBA and FBD. However, disparities persisted, with LICs showing lower coverage and greater variability, especially for ANC4+. Higher MHS coverage, particularly ANC4+ and FBD, was associated with lower MMR, with stronger effects among more educated women.

### Trends in MMR and MHS in LMICs

The trends observed in this study indicate an overall decline in median MMR in LMICs between 2015 and 2023. Our findings are consistent with long-standing evidence that global commitments to improving maternal health, beginning during the Millennium Development Goals era and continuing under the SDGs, have contributed to improved maternal survival [1,10,16,32,33]. The continued decline likely reflects sustained investments in MHS [10,18,34], expansion of essential obstetric care [18], and strengthened maternal health policies implemented across many countries [34–37]. The temporary increase in MMR observed in 2021, however, highlights the fragility of maternal health gains in the face of global pandemics. Evidence from the COVID-19 pandemic indicates that disruptions to maternal health services and access to care adversely affected maternal health outcomes and may have contributed to the reversal in progress observed during this period [10,38,39]. Despite the overall decline, the median MMR remains above the SDG 3.1 target, indicating that substantial progress is still needed to reduce maternal mortality across LMICs.

Nevertheless, disparities across income groups persist. Although MMR declined across all income groups, the burden remains concentrated in LICs. Similar patterns have been widely documented, with socioeconomic conditions and health system capacity playing a vital role in shaping maternal health outcomes across countries [10,11,21,25,40]. Inequalities in access to MHS coverage are also well established, particularly among disadvantaged populations and resource-constrained settings [41–46]. Reductions in maternal mortality often occur in phases, from expanded access to improvements in service quality, referral capacity, and broader health system performance [18,40,41,46–53]. The persistence of relatively high levels of MMR in several LMICs, particularly in LICs, may reflect broader contextual and systemic constraints [10,18,40–42,54,55].

Consistent with these disparities in maternal mortality, our findings also reveal important patterns in MHS coverage across LMICs. Although coverage improved across all income groups from 2015 to 2023, particularly for SBA and FBD, levels remained substantially lower in LICs, especially for ANC4+, compared with higher-income groups. These differences likely contribute to the uneven progress in reducing maternal mortality. While access to key MHS has expanded in many settings, disparities in service coverage have remained evident over time [18,41–43,56].

Country-level variation further highlights these differences. While several countries made progress toward the SDG targets, others showed slower progress or even increases in MMR. Similar divergence across LMICs has been widely documented and may be influenced by structural differences in health system capacity [18,57,58], conflict [18,59,60], economic instability [18,47,59,61], governance and policy environments [18,22,59,62,63], and geographic disparities in access to essential MHS [16,18,22]. These contextual factors may partly account for the substantial heterogeneity observed across countries, including the contrasting trends reported for Sudan, Eswatini, Belarus, and Venezuela. For example, conflict-related disruptions in Sudan [64] and prolonged economic instability in Venezuela, accompanied by population displacement [65], may have affected access to MHS and maternal health outcomes. In addition, differences in data availability and quality may have influenced maternal mortality estimates across countries [3,11,12]. Health system quality and readiness have also been increasingly recognised as key determinants of maternal health outcomes in many LMIC settings [46,54,55]. Overall, progress remains uneven, and substantial efforts are still needed to accelerate MMR reductions across many LMICs.

### Association between MHS coverage and MMR in LMICs

Higher coverage of key MHS, particularly ANC4+ and FBD, was associated with lower maternal mortality across LMICs. Adequate ANC enables early detection and management of pregnancy-related risks and promotes continuity of care across the maternal health continuum [10,18,32,40,66–68]. Evidence from LMICs, including the Philippines [22], India [21], and China [69], shows that expanded ANC coverage is associated with reduced maternal mortality and improved maternal health outcomes. FBD also plays a critical role in preventing intrapartum and early postpartum deaths by ensuring access to SBA and emergency obstetric care (EmOC) [10,40,68,70–72]. Women receiving adequate ANC are more likely to deliver in health facilities, highlighting the complementary roles of these services [67,71,73]. Strengthening access to these services remains fundamental to accelerating progress toward the SDG target.

The association between MHS coverage and MMR also differed across income groups. Although the largest absolute reductions in MMR occurred in LICs, relative declines were greater in middle-income countries. These patterns may reflect differences in baseline maternal mortality levels and health system development [3,11,18,40,50]. One possible explanation is that gains in coverage may translate more efficiently into lower MMR in settings with stronger health systems, whereas resource limitations may constrain these benefits in lower-income settings. Factors such as shortages of skilled health workers, limited referral capacity, and gaps in service quality may contribute to these differences [10,40,41,46,50,51,55].

Health system capacity further shapes the extent to which MHS translates into improved outcomes. We found that higher nurse, physician, and midwife densities were associated with lower maternal mortality across LMICs. A well-distributed health workforce improves access, continuity, and quality of care, particularly in resource-limited settings [32,45,48,74–76]. Adequate workforce capacity also strengthens referral systems and the provision of EmOC, both of which are essential for managing complications during pregnancy and childbirth [32,45,48,53,72,77]. Strengthening workforce capacity, therefore, remains a key strategy for improving maternal outcomes.

In contrast, not all health system indicators demonstrated independent associations with MMR. CHE was not independently associated with MMR after adjustment. Although previous studies have linked higher health expenditure to improved maternal health outcomes [18,31,36,41], CHE represents overall health spending and may not adequately capture how resources are allocated to MHS. Consequently, expenditure levels alone may not fully explain variations in MMR across LMICs.

Maternal education also modifies the association between ANC4+ coverage and MMR. The interaction between maternal education and ANC4+ indicates that this association varies by education level. Higher ANC4+ coverage was more strongly associated with lower MMR among women with higher education. Maternal education improves health literacy, care-seeking behaviour, and recognition of pregnancy-related complications, enabling more effective use of MHS [45,51,78–82]. Although the magnitude of the interaction was modest, it suggests that improvements in ANC4+ coverage alone may not yield comparable reductions in MMR across populations with different levels of maternal education. Stratified analysis further showed stronger associations among women with moderate to high levels of maternal education in UMCs, whereas associations were weaker in lower-income settings and among less educated women. These findings suggest that effectiveness depends not only on service availability but also on broader socioeconomic conditions such as education that influences utilisation [56,78,80,81].

Our results also indicate that while ANC4+ and FBD were significantly associated with lower maternal mortality, SBA alone was not independently associated with MMR after adjustment. This contrasts with a substantial body of evidence identifying SBA as a key determinant of maternal survival [10,32,83,84]. The absence of an independent association may reflect the inability of SBA coverage to capture differences in the quality of care provided at delivery, as well as the relatively high levels of SBA coverage observed across many LMICs. Multicollinearity is unlikely to explain this finding, as diagnostic testing indicated no evidence of severe collinearity among the MHS indicators (Table S1 in the **Online Supplementary Document**). Moreover, its effectiveness may also depend on functioning referral systems, adequate health infrastructure, and the overall quality of EmOC [46–48]. Therefore, ensuring that SBA is supported by well-functioning health systems is essential.

### Limitations and strengths

This study has several limitations. Because we relied on country-level aggregated data, our analysis reflects population-level relationships and cannot establish individual-level associations, thereby making it subject to the ecological fallacy and limited causal inference. We categorised countries according to their 2015 World Bank income classification to facilitate consistent comparisons over time. While 28 countries experienced economic transitions during the study period, our analysis relied strictly on baseline income status, which may introduce some misclassification bias.

Unfortunately, differences in data quality across countries are unavoidable, and imputing missing values with the most recent available value may not fully capture temporal changes in indicators with a substantial lack of data, thereby introducing a ‘flat line’ bias rather than capturing the actual rate of change. For instance, we did not include PNC in the primary analyses because of substantial missing data across countries and years, and since PNC is closely linked to maternal outcomes, its omission may have inflated the observed association between MHS coverage and MMR, whereas these effects are partially driven by PNC.

Coverage indicators measure service utilisation but do not capture the quality, timing, or effectiveness of care. We assessed antenatal care using ANC4+, the indicator available from the WHO GHO for cross-country comparison during the study period [27]. Although the WHO currently recommends eight antenatal visits, comparable ANC8+ data remained limited across countries and throughout the study period. Future studies should incorporate ANC8+ coverage as more comprehensive data become available.

We measured maternal education using female literacy among women aged 15 years and older. While literacy is strongly associated with educational attainment, it may not fully capture differences in years of schooling or educational level achieved, and should be interpreted as a proxy measure of maternal education.

We did not directly measure several contextual factors discussed as potential explanations for differences in MMR across countries and income groups, including health system capacity, referral systems, service quality, governance, and conflict. Accordingly, such factors should be interpreted as plausible contextual explanations for the observed patterns rather than as mechanisms directly evaluated in the present analysis. In addition, we did not design the study to evaluate whether countries are on track to achieve the SDG 3.1 target by 2030. Although we examined trends in MMR and MHS during the SDG period, a formal assessment of future target attainment would require projection analyses that are beyond the scope of the present study. Estimates derived from exponentiated regression coefficients should be interpreted with caution, particularly in stratified analyses, where model complexity, limited variability, and data sparsity may affect estimation stability. For example, one subgroup model for women with low education in UMCs did not converge because only a single observation was available; consequently, no estimate could be obtained for that subgroup.

Despite these limitations, the study has several strengths. The use of recent longitudinal data allows a comprehensive assessment of trends in MMR and MHS coverage during the SDG period. Furthermore, the inclusion of multiple service, health system, and socioeconomic indicators analysed using a population-weighted GLIMMIX with a gamma-log link function appropriately accounts for outcome skewness and cross-country heterogeneity.

### Policy implications

Progress toward the SDGs, particularly the target of reducing maternal mortality under SDG 3.1, depends not only on expanding MHS coverage but also on strengthening health systems and addressing persistent inequalities. Although MHS utilisation increased across LMICs, the findings suggest that expanding coverage alone may be insufficient to sustain reductions in maternal mortality. Improvements in ANC4+ and FBD were associated with lower maternal mortality; however, their effectiveness may be modified by maternal education. This suggests that efforts to increase service coverage should be accompanied by targeted interventions that improve women’s educational opportunities and health literacy to maximise the benefits of MHS [78,79,82]. Previous studies have highlighted the importance of factors including the availability of skilled health personnel, health system readiness, and quality of care in shaping maternal health outcomes [18,24,40,49,54]. These findings suggest that expanding coverage alone is insufficient unless improvements in service quality and health system capacity occur simultaneously.

Sustained progress, therefore, requires strengthening the foundations of the health system while advancing toward UHC (SDG 3.8) [1,9,18]. Policy efforts should prioritise improving the quality and readiness of MHS, strengthening workforce capacity, and ensuring that referral systems for EmOC are functional. [46,47,52,53,67,72,74,77] Persistent differences in MHS coverage across income groups further indicate that inequalities in access remain a major challenge. Addressing these gaps, particularly in LICs and resource-limited settings, is essential for reducing inequalities in maternal survival across and within countries, in line with SDG 10. [41–46,56]

## CONCLUSIONS

This comprehensive multi-country analysis shows that MMR declined across LMICs between 2015 and 2023; however, MMRs remain above the SDG 3.1 target in many settings. Higher coverage of ANC4+ and FBD, as well as greater densities of skilled health personnel and higher maternal education, were consistently associated with lower MMR across countries and income groups. Continued progress in reducing maternal mortality will require strengthening MHS, enhancing health system capacity, and addressing persistent inequalities shaping maternal health outcomes across LMICs.

## Additional material


Online Supplementary Document

## Data Availability

**Data availability:** The aggregated raw data used in this study are available from the corresponding author upon reasonable request.
