# Supplementary material for: Tracking the impact of maternal health service coverage on maternal mortality across 137 low- and middle-income countries in the progress toward Sustainable Development Goals
Source: J Glob Health. 2026 Oct 2;16:04324. doi: 10.7189/jogh.16.04324 (PMC13631974; doi:10.7189/jogh.16.04324)
Supplement: Supplementary file 1 [file jogh-16-04324-s001.pdf]

Supplement to: Ganancial FS, Hsieh VCR. Tracking the impact of maternal health service coverage on maternal mortality across 137 low- and middle-income countries in the progress toward sustainable development goals. J Glob Health. 2026;16:04324.

Supplementary materials (Tables)

Table S1. Missing observations and imputation across all study variables, 2015-2023

No.	Variable	N	2015-2023	
			**no. of missing**	**% of missing**
**1**	Maternal mortality ratio	3288	198	6.02
**2**	Antenatal care, at least four visits	3288	1026	31.20
**3**	Births attended by skilled professionals	3288	902	27.43
**4**	Institutional delivery	3288	967	29.41
**5**	Postpartum care	3288	1124	34.18
**6**	Density of Nurses and Midwives	3288	605	18.40
**7**	Density of physicians	3288	628	19.10
**8**	Out-of-pocket expenditure	3288	319	9.70
**9**	Current Health Expenditure	3288	319	9.70
**10**	Maternal education	3288	972	29.56

Table S2. Multicollinearity diagnostics for maternal mortality ratio predictors

Variable	Tolerance	VIF	Interpretation
Antenatal care, at least four visits	0.37	2.69	Moderate multicollinearity
Births attended by skilled health personnel	0.25	3.93	Moderate multicollinearity
Institutional delivery	0.22	4.49	Moderate multicollinearity
Density of nurses and midwives	0.36	2.74	Moderate multicollinearity
Density of physicians	0.40	2.49	No serious multicollinearity
Out-of-pocket expenditure	0.81	1.23	No serious multicollinearity
Current health expenditure	0.90	1.11	No serious multicollinearity
Maternal education	0.33	3.00	Moderate multicollinearity
**Dependent variable: Maternal mortality ratio**			

VIF – variance inflation factors

Table S3. Sample distribution and list of countries per 2015 World Bank income classification, N=137

**Low-income countries**[a] **(n=31)**	**Lower-middle countries**[b] **(n=51)**	**Upper-middle countries**[c] **(n=55)**
Afghanistan	Armenia	Albania
Benin	Bangladesh	Algeria
Burkina Faso	Bhutan	Angola
Burundi	Bolivia	Argentina
Central African Republic	Cabo Verde	Azerbaijan
Chad	Cambodia	Belarus
Comoros	Cameroon	Belize
Congo, Dem. Rep.	Congo, Rep.	Bosnia and Herzegovina
Dem. People's Rep. of Korea	Cote d'Ivoire	Botswana
Eritrea	Djibouti	Brazil
Ethiopia	Egypt	Bulgaria
Gambia	El Salvador	China
Guinea	Eswatini	Colombia
Guinea-Bissau	Ghana	Costa Rica
Haiti	Guatemala	Cuba
Liberia	Honduras	Dominica
Madagascar	India	Dominican Republic
Malawi	Indonesia	Ecuador
Mali	Kenya	Equatorial Guinea
Mozambique	Kiribati	Fiji
Nepal	Kyrgyz Republic	Gabon
Niger	Lao PDR	Georgia
Rwanda	Lesotho	Grenada
Senegal	Mauritania	Guyana
Sierra Leone	Micronesia	Iran
Somalia	Mongolia	Iraq
South Sudan	Morocco	Jamaica
Togo	Myanmar	Jordan
Uganda	Nicaragua	Kazakhstan
United Rep. of Tanzania	Nigeria	Lebanon
Zimbabwe	Pakistan	Libya
	Papua New Guinea	Malaysia
	Philippines	Maldives
	Republic of Moldova	Marshall Islands
	Samoa	Mauritius
	Sao Tome and Principe	Mexico
	Solomon Islands	Montenegro
	Sri Lanka	Namibia
	Sudan	North Macedonia
	Syrian Arab Republic	Palau
	Tajikistan	Panama
	Timor-Leste	Paraguay
	Tonga	Peru
	Tunisia	Romania

Low-income countries[a] (n=31)	Lower-middle countries[b] (n=51)	Upper-middle countries[c] (n=55)
	Ukraine	Russian Federation
	Uzbekistan	Saint Lucia
	Vanuatu	St. Vincent and the Grenadines
	Viet Nam	Serbia
	West Bank and Gaza	South Africa
	Yemen	Suriname
	Zambia	Thailand
		Turkiye
		Turkmenistan
		Tuvalu
		Venezuela

[a] countries with gross national income of 1045 USD or less
[b] countries with gross national income of 1046 to 4125 USD
[c] countries with gross national income of 4126 to 12,735 USD

Table S4. Changes in World Bank income classification between 2015 and 2023

Country	2015 income classification	2023 income classification*
Benin	Low-income	Lower-middle income
Comoros	Low-income	Lower-middle income
Guinea	Low-income	Lower-middle income
Haiti	Low-income	Lower-middle income
Nepal	Low-income	Lower-middle income
Senegal	Low-income	Lower-middle income
Tanzania	Low-income	Lower-middle income
Zimbabwe	Low-income	Lower-middle income
Armenia	Lower-middle income	Upper-middle income
El Salvador	Lower-middle income	Upper-middle income
Indonesia	Lower-middle income	Upper-middle income
Moldova	Lower-middle income	Upper-middle income
Mongolia	Lower-middle income	Upper-middle income
Sudan	Lower-middle income	Low-income
Syrian Arab Republic	Lower-middle income	Low-income
Tonga	Lower-middle income	Upper-middle income
Ukraine	Lower-middle income	Upper-middle income
Yemen	Lower-middle income	Low-income
Angola	Upper-middle income	Lower-middle income
Bulgaria	Upper-middle income	High-income
Guyana	Upper-middle income	High-income
Jordan	Upper-middle income	Lower-middle income
Lebanon	Upper-middle income	Lower-middle income
Palau	Upper-middle income	High-income
Panama	Upper-middle income	High-income
Romania	Upper-middle income	High-income
Russian Federation	Upper-middle income	High-income

Legend:

Low-income: countries with gross national income of 1,145 USD or less
Lower-middle income: countries with gross national income of 1,146 to 4,515 USD
Upper-middle income: countries with gross national income of 4,516 to 14,005 USD
High-income countries: countries with gross national income of above 14,005 USD

Table S5. Trends in maternal mortality ratio (MMR) in low- and middle-income countries (LMICs), 2015-2023

Country	2015 (a)	2023 (b)	Absolute change (b-a)	% of change	AAPC
Low income (n=31)					
Mozambique	216.1	99.2	-116.9	-54.1	-9.3
South Sudan	1436.0	691.8	-744.2	-51.8	-8.7
Sierra Leone	730.1	353.6	-376.5	-51.6	-8.7
Ethiopia	400.7	194.9	-205.8	-51.4	-8.6
Uganda	299.2	170.3	-128.9	-43.1	-6.8
Nepal	235.6	142.0	-93.6	-39.7	-6.1
Chad	1176.0	747.5	-428.5	-36.4	-5.5
Malawi	353.8	225.0	-128.8	-36.4	-5.5
United Republic of Tanzania	433.2	275.8	-157.4	-36.3	-5.5
Senegal	356.7	237.4	-119.3	-33.5	-5.0
Gambia	526.7	354.4	-172.3	-32.7	-4.8
Afghanistan	741.2	520.5	-220.7	-29.8	-4.3
Central African Republic	980.5	691.7	-288.8	-29.5	-4.3
Eritrea	412.8	291.4	-121.4	-29.4	-4.3
Somalia	784.2	562.6	-221.6	-28.3	-4.1
Burkina Faso	327.8	241.8	-86.0	-26.2	-3.7
Niger	473.0	350.3	-122.7	-25.9	-3.7
Togo	470.4	348.9	-121.5	-25.8	-3.7
Burundi	525.2	392.0	-133.2	-25.4	-3.6
Mali	489.7	367.3	-122.4	-25.0	-3.5
Comoros	236.6	178.9	-57.7	-24.4	-3.4
Guinea	641.7	494.1	-147.6	-23.0	-3.2
Congo, Dem. Rep.	545.4	427.0	-118.4	-21.7	-3.0
Rwanda	291.6	229.5	-62.1	-21.3	-3.0
Guinea-Bissau	631.2	505.4	-125.8	-19.9	-2.7
Liberia	784.2	627.7	-156.5	-20.0	-2.7
Zimbabwe	427.9	357.6	-70.3	-16.4	-2.2
Benin	606.4	518.2	-88.2	-14.5	-2.0
Haiti	363.7	327.6	-36.1	-9.9	-1.3
Democratic People's Republic of Korea	71.9	66.9	-5.0	-6.9	-0.9
Madagascar	473.9	445.4	-28.5	-6.0	-0.8
Lower-middle income (n=51)					
Eswatini	283.0	117.9	-165.1	-58.3	-10.4
Zambia	154.4	85.5	-69.0	-44.7	-7.1
Bangladesh	203.5	115.1	-88.4	-43.4	-6.9
São Tomé and Príncipe	127.8	75.4	-52.4	-41.0	-6.4
Cameroon	417.3	258.2	-159.1	-38.1	-5.8
Lao, PDR	179.6	112.2	-67.4	-37.5	-5.7
India	128.5	80.5	-48.0	-37.4	-5.7
West Bank and Gaza	25.7	16.1	-9.6	-37.3	-5.7
Timor-Leste	305.0	192.4	-112.6	-36.9	-5.6
Bhutan	68.4	46.7	-21.7	-31.8	-4.7
Tajikistan	20.2	13.9	-6.4	-31.5	-4.6
Egypt	24.3	17.0	-7.3	-30.2	-4.4
Morocco	99.7	69.8	-29.9	-30.0	-4.4

Country	2015 (a)	2023 (b)	Absolute change (b-a)	% of change	AAPC
Micronesia	181.9	128.8	-53.1	-29.2	-4.2
Cote d'Ivoire	505.1	359.0	-146.1	-28.9	-4.2
Kyrgyz Republic	57.7	41.9	-15.8	-27.4	-3.9
Kenya	202.8	148.9	-53.9	-26.6	-3.8
Indonesia	190.2	140.5	-49.7	-26.1	-3.7
Nicaragua	80.7	60.4	-20.3	-25.2	-3.6
Ghana	310.3	234.3	-76.0	-24.5	-3.5
Pakistan	202.3	155.0	-47.3	-23.4	-3.3
Djibouti	210.1	161.9	-48.2	-22.9	-3.2
Armenia	25.1	19.5	-5.6	-22.2	-3.1
Mongolia	51.9	40.5	-11.4	-21.9	-3.0
Lesotho	593.9	478.0	-115.9	-19.5	-2.7
Philippines	103.5	83.8	-19.8	-19.1	-2.6
Mauritania	469.2	381.1	-88.1	-18.8	-2.6
Honduras	57.1	46.8	-10.4	-18.1	-2.5
Viet Nam	57.6	47.6	-10.0	-17.4	-2.4
Cambodia	165.8	137.4	-28.4	-17.1	-2.3
Myanmar	221.5	184.6	-36.9	-16.7	-2.3
Sri Lanka	22.0	18.3	-3.6	-16.5	-2.2
Guatemala	112.0	93.7	-18.3	-16.3	-2.2
Syrian Arab Republic	24.3	20.4	-3.9	-16.2	-2.2
Solomon Islands	146.0	122.6	-23.4	-16.0	-2.2
Tonga	78.8	66.8	-12.0	-15.3	-2.1
Congo, Rep.	284.1	241.1	-43.0	-15.1	-2.0
Uzbekistan	31.2	26.5	-4.7	-15.1	-2.0
Nigeria	1168.0	992.8	-175.2	-15.0	-2.0
Papua New Guinea	220.7	188.6	-32.1	-14.5	-2.0
Sudan	297.9	255.7	-42.2	-14.2	-1.9
Cabo Verde	46.4	40.0	-6.3	-13.7	-1.8
Ukraine	17.3	15.0	-2.3	-13.5	-1.8
Yemen	134.7	118.4	-16.3	-12.1	-1.6
Bolivia	166.1	146.4	-19.7	-11.9	-1.6
Kiribati	90.4	79.8	-10.6	-11.8	-1.6
Tunisia	40.3	35.7	-4.6	-11.3	-1.5
El Salvador	43.0	39.1	-3.8	-8.9	-1.2
Vanuatu	109.3	100.0	-9.3	-8.5	-1.1
Republic of Moldova	20.4	18.7	-1.7	-8.4	-1.1
Samoa	107.7	101.5	-6.2	-5.8	-0.7
Upper-middle income (n=55)					
Namibia	306.6	138.9	-167.7	-54.7	-9.4
Dominica	62.8	35.6	-27.2	-43.4	-6.9
China	26.2	16.3	-9.9	-37.9	-5.8
Russian Federation	14.8	9.4	-5.4	-36.4	-5.5
Guyana	117.2	74.7	-42.5	-36.2	-5.5
Romania	17.7	11.7	-6.0	-33.8	-5.0
Georgia	29.6	19.7	-9.9	-33.5	-5.0
Iran	22.2	15.8	-6.4	-28.7	-4.1
Peru	71.5	51.5	-20.0	-28.0	-4.0
North Macedonia	3.8	2.8	-1.1	-27.9	-4.0

Country	2015 (a)	2023 (b)	Absolute change (b-a)	% of change	AAPC
Suriname	115.1	83.6	-31.5	-27.3	-3.9
Angola	249.4	183.2	-66.2	-26.5	-3.8
Jordan	41.2	30.8	-10.4	-25.3	-3.6
Turkmenistan	6.1	4.5	-1.5	-25.3	-3.6
Kazakhstan	12.8	9.6	-3.2	-25.0	-3.5
Albania	9.1	6.9	-2.3	-24.8	-3.5
Belarus	1.5	1.1	-0.4	-24.7	-3.5
Turkiye	19.4	14.6	-4.8	-24.5	-3.5
Bulgaria	7.3	5.6	-1.7	-23.4	-3.3
Iraq	85.9	66.0	-19.9	-23.2	-3.2
Panama	48.1	37.0	-11.1	-23.0	-3.2
Maldives	40.9	31.8	-9.1	-22.2	-3.1
Equatorial Guinea	222.2	173.7	-48.5	-21.8	-3.0
Libya	76.1	59.5	-16.6	-21.8	-3.0
South Africa	150.4	117.6	-32.8	-21.8	-3.0
Algeria	79.5	62.5	-17.0	-21.4	-3.0
Serbia	13.3	10.7	-2.5	-19.1	-2.6
Fiji	37.1	30.2	-6.8	-18.5	-2.5
Azerbaijan	21.4	17.9	-3.5	-16.4	-2.2
Cuba	41.5	34.8	-6.7	-16.1	-2.2
Montenegro	7.0	5.8	-1.1	-16.1	-2.2
Marshall Islands	183.8	154.8	-29.0	-15.8	-2.1
Colombia	70.3	59.4	-10.9	-15.5	-2.1
Argentina	38.6	33.2	-5.4	-14.0	-1.9
Ecuador	64.2	55.4	-8.9	-13.8	-1.8
Malaysia	29.8	26.4	-3.4	-11.5	-1.5
Paraguay	63.7	58.3	-5.4	-8.5	-1.1
Bosnia and Herzegovina	6.7	6.2	-0.5	-7.4	-1.0
Saint Vincent and the Grenadines	59.2	55.8	-3.4	-5.7	-0.7
Costa Rica	25.5	24.1	-1.4	-5.6	-0.7
Saint Lucia	45.8	44.0	-1.9	-4.0	-0.5
Gabon	239.1	232.7	-6.4	-2.7	-0.3
Mexico	43.2	42.1	-1.1	-2.4	-0.3
Tuvalu	169.0	169.7	0.7	0.4	0.1
Lebanon	14.9	15.0	0.1	0.7	0.1
Thailand	33.7	34.5	0.7	2.2	0.3
Grenada	46.2	48.3	2.0	4.4	0.5
Brazil	63.0	66.9	4.0	6.3	0.8
Mauritius	61.5	65.9	4.4	7.2	0.9
Botswana	140.5	155.1	14.6	10.4	1.2
Palau	78.7	89.4	10.7	13.6	1.6
Dominican Republic	102.3	124.5	22.2	21.7	2.5
Jamaica	100.3	130.1	29.8	29.7	3.3
Belize	47.2	66.8	19.7	41.7	4.5
Venezuela	136.3	226.7	90.4	66.3	6.6

AAPC – average annual percent change

Table S6. Descriptive statistics of maternal mortality ratio (MMR) and maternal health-related indicators in low- and middle-income countries by World Bank income group, 2015-2023

**MHS indicator**	**Overall (n=137)**															
	**2015**								**2023**							
	**Obs**	**Missing**	**Median**	**Q1**	**Q3**	**IQR**	**Min.**	**Max.**	**Obs**	**Missing**	**Median**	**Q1**	**Q3**	**IQR**	**Min.**	**Max.**
Maternal mortality ratio	137	0	109.3	41.5	297.9	256.4	1.5	1436.0	137	0	85.5	35.6	225.0	189.5	1.1	992.8
Maternal health services																
Antenatal care visits, at least four (%)	128	9	75.0	55.1	87.1	32.0	6.3	99.7	132	5	77.8	61.2	88.4	27.3	17.0	99.9
Births attended by skilled health personnel (%)	137	0	91.0	68.0	99.0	31.0	9.0	100.0	137	0	96.0	83.0	100.0	17.0	32.0	100.0
Institutional delivery (%)	133	4	87.4	63.0	98.0	35.0	9.4	100.0	134	3	93.0	80.1	98.6	18.5	11.5	100.0
Health system factors																
Nurses and midwives (per 10 000 population)	134	3	16.9	8.0	37.3	29.3	1.3	113.6	137	0	22.2	11.1	42.0	31.0	1.7	110.8
Physicians (per 10 000 population)	135	2	6.7	1.7	18.1	16.3	0.1	78.0	137	0	7.6	2.2	20.4	18.3	0.4	95.4
OOP expenditure (% of CHE)	135	2	35.4	22.0	49.4	27.4	0.3	81.6	137	0	33.9	21.9	45.7	23.9	0.4	79.2
Socioeconomic factors																
Maternal education	127	10	85.0	55.2	95.0	39.8	13.9	100.0	130	7	89.0	66.5	96.0	29.5	18.9	100.0
CHE, % of GDP	135	2	5.3	4.1	7.3	3.2	1.8	19.7	137	0	5.7	4.2	7.9	3.7	2.0	23.1

CHE – current health expenditure, GDP – gross domestic product, IQR – interquartile range, Max – maximum, MHS – maternal health services; Min – minimum, Obs – observations, OOP – out-of-pocket, Q1 – first quartile, Q3 – third quartile

Table S6. Continued

MHS indicator	Low income (n=31)															
	2015								2023							
	Obs	Missing	Median	Q1	Q3	IQR	Min.	Max.	Obs	Missing	Median	Q1	Q3	IQR	Min.	Max.
Maternal mortality ratio	31	0	473.0	353.8	641.7	287.9	71.9	1436.0	31	0	353.6	229.5	505.4	275.9	66.9	747.5
Maternal health services																
Antenatal care visits, at least four (%)	31	0	48.0	38.1	59.5	21.4	6.3	93.5	31	0	56.7	43.0	71.5	28.5	17.0	93.7
Births attended by skilled health professionals (%)	31	0	53.0	44.0	78.0	34.0	9.0	100.0	31	0	75.0	50.0	88.0	38.0	32.0	100.0
Institutional delivery (%)	31	0	57.4	40.3	76.2	35.9	9.4	94.7	31	0	73.4	50.4	85.5	35.1	11.5	96.7
Health system factors																
Nurses and midwives (per 10 000 population)	30	1	4.9	2.6	9.1	6.5	1.3	40.7	31	0	7.9	4.9	11.7	6.7	1.7	43.8
Physicians (per 10 000 population)	30	1	0.7	0.5	1.1	0.6	0.1	34.5	31	0	1.4	0.9	2.1	1.3	0.4	36.3
OOP expenditure (% of CHE)	30	1	37.5	25.8	51.4	25.6	11.0	78.4	31	0	42.5	28.7	54.3	25.6	8.2	78.0
Socioeconomic factors																
Maternal education	30	1	42.3	24.4	55.2	30.8	13.9	100.0	31	0	49.0	31.3	69.4	38.1	18.9	100.0
CHE, % of GDP	30	1	5.1	4.1	6.6	2.5	2.7	19.7	31	0	4.5	3.6	8.1	4.5	2.6	23.1

CHE – current health expenditure, GDP – gross domestic product, IQR – interquartile range, Max – maximum, MHS – maternal health services; Min – minimum, Obs – observations, OOP – out-of-pocket, Q1 – first quartile, Q3 – third quartile

Table S6. Continued

MHS indicator	Lower-middle income (n=51)															
	2015								2023							
	Obs	Missing	Median	Q1	Q3	IQR	Min.	Max.	Obs	Missing	Median	Q1	Q3	IQR	Min.	Max.
Maternal mortality ratio	51	0	127.8	51.9	210.1	158.2	17.3	1168.0	51	0	93.7	40.5	155.0	114.5	13.9	992.8
Maternal health services																
Antenatal care visits, at least four (%)	48	3	72.2	55.0	84.6	29.6	25.1	95.5	49	2	79.0	60.9	88.3	27.4	25.7	96.0
Births attended by skilled health professionals (%)	51	0	87.0	69.0	98.0	29.0	29.0	100.0	51	0	91.0	80.0	98.0	18.0	44.0	100.0
Institutional delivery (%)	50	1	78.5	61.3	91.0	29.7	22.1	99.5	51	0	88.5	78.2	97.0	18.8	27.7	100.0
Health system factors																
Nurses and midwives (per 10 000 population)	49	2	14.2	9.3	22.1	12.8	2.6	113.6	51	0	18.0	10.8	32.4	21.6	1.9	110.8
Physicians (per 10 000 population)	50	1	4.7	2.0	9.1	7.1	0.5	69.8	51	0	6.8	2.6	12.8	10.2	0.6	41.3
OOP expenditure (% of CHE)	51	0	42.7	19.0	55.7	36.7	1.1	81.6	51	0	34.6	21.9	51.1	29.2	0.8	79.1
Socioeconomic factors																
Maternal education	48	3	81.5	62.5	92.0	29.5	35.0	100.0	49	2	86.0	70.0	95.0	25.0	40.3	100.0
CHE, % of GDP	51	0	4.7	3.7	7.2	3.5	1.8	12.7	51	0	5.3	4.3	8.1	3.8	2.0	14.3

CHE – current health expenditure, GDP – gross domestic product, IQR – interquartile range, Max – maximum, MHS – maternal health services; Min – minimum, Obs – observations, OOP – out-of-pocket, Q1 – first quartile, Q3 – third quartile

Table S6. Continued

MHS indicator	Upper-middle income (n=55)															
	2015								2023							
	**Obs**	**Missing**	**Median**	**Q1**	**Q3**	**IQR**	**Min.**	**Max.**	**Obs**	**Missing**	**Median**	**Q1**	**Q3**	**IQR**	**Min.**	**Max.**
Maternal mortality ratio	55	0	46.2	21.4	85.9	64.5	1.5	306.6	55	0	42.1	15.8	74.7	58.9	1.1	232.7
Maternal health services																
Antenatal care visits, at least four (%)	49	6	86.6	77.4	93.6	16.2	49.6	99.7	52	3	84.7	75.6	92.8	17.2	60.3	99.9
Births attended by skilled health professionals (%)	55	0	99.0	94.0	100.0	6.0	47.0	100.0	55	0	99.0	97.0	100.0	3.0	50.0	100.0
Institutional delivery (%)	52	3	98.2	94.4	99.6	5.2	45.8	100.0	52	3	98.6	94.8	99.6	4.8	45.6	100.0
Health system factors																
Nurses and midwives (per 10 000 population)	55	0	31.7	19.3	60.2	41.0	3.0	109.5	55	0	40.5	27.4	59.7	32.3	13.8	108.2
Physicians (per 10 000 population)	55	0	18.0	9.1	23.4	14.3	1.3	78.0	55	0	20.4	11.6	29.4	17.9	1.5	95.4
OOP expenditure (% of CHE)	54	1	32.4	21.0	40.6	19.6	0.3	76.2	55	0	27.7	18.6	38.9	20.3	0.4	79.2
Socioeconomic factors																
Maternal education	49	6	93.0	91.0	98.0	7.0	51.9	100.0	50	5	95.0	90.8	98.3	7.5	62.5	100.0
CHE, % of GDP	54	1	5.8	4.5	7.5	3.0	2.3	14.9	55	0	5.8	4.6	7.7	3.2	2.9	18.4

CHE – current health expenditure, GDP – gross domestic product, IQR – interquartile range, Max – maximum, MHS – maternal health services; Min – minimum, Obs – observations, OOP – out-of-pocket, Q1 – first quartile, Q3 – third quartile

Table S7. Country-level trends in at least four antenatal care (ANC4+) coverage, 2015-2023

Country	2015 (a)	2023 (b)	Absolute change (b-a)	% of change	AAPC
Low income (n=31)					
Somalia	6.3	24.4	18.1	287.3	18.4
Afghanistan	17.8	33.4	15.6	87.6	8.2
Burkina Faso	47.2	72.1	24.9	52.8	5.4
United Republic of Tanzania	42.8	65.1	22.3	52.1	5.4
Senegal	46.7	68.4	21.7	46.5	4.9
Nepal	59.5	80.5	21.0	35.3	3.9
Ethiopia	32.1	43.0	10.9	34.0	3.7
Comoros	48.9	64.3	15.4	31.5	3.5
Guinea-Bissau	64.9	80.7	15.8	24.4	2.8
Uganda	47.6	56.7	9.1	19.1	2.2
Mali	38.0	44.7	6.7	17.6	2.1
Madagascar	51.1	59.9	8.8	17.2	2.0
Congo, Dem. Rep.	48.0	56.0	8.0	16.7	2.0
Malawi	44.7	50.5	5.8	13.0	1.5
Central African Republic	38.1	41.4	3.3	8.7	1.0
Rwanda	43.9	47.2	3.3	7.5	0.9
Liberia	78.1	83.8	5.7	7.3	0.9
Burundi	33.4	34.9	1.5	4.5	0.6
Sierra Leone	76.0	78.8	2.8	3.7	0.5
Guinea	56.6	58.3	1.7	3.0	0.4
Gambia	77.6	78.5	0.9	1.2	0.1
Democratic People’s Republic of Korea	93.5	93.7	0.2	0.2	0.0
Chad	31.0	31.0	0.0	0.0	0.0
Eritrea	57.4	57.4	0.0	0.0	0.0
South Sudan	17.0	17.0	0.0	0.0	0.0
Haiti	67.3	66.6	-0.7	-1.0	-0.1
Niger	38.5	37.3	-1.2	-3.1	-0.4
Togo	57.2	54.8	-2.4	-4.2	-0.5
Zimbabwe	75.7	71.5	-4.2	-5.6	-0.7
Mozambique	51.7	48.6	-3.1	-6.0	-0.8
Benin	58.7	52.6	-6.1	-10.4	-1.4
Lower-middle income (n=51)					
Lao PDR	36.9	62.2	25.3	68.6	6.7
Pakistan	36.6	52.4	15.8	43.2	4.6
Timor-Leste	55.1	76.7	21.6	39.2	4.2
Bolivia	58.8	81.3	22.5	38.3	4.1
Bangladesh	31.2	40.5	9.3	29.8	3.3
Yemen	25.1	32.4	7.3	29.1	3.2
India	45.4	58.5	13.1	28.9	3.2
Nicaragua	72.0	91.9	19.9	27.6	3.1
Cote d'Ivoire	44.2	56.3	12.1	27.4	3.1
Tonga	70.4	88.5	18.1	25.7	2.9
Tajikistan	52.5	64.2	11.7	22.3	2.6
Viet Nam	73.7	88.2	14.5	19.7	2.3
Cabo Verde	72.3	85.6	13.3	18.4	2.1
Nigeria	51.1	60.4	9.3	18.2	2.1
El Salvador	74.0	87.2	13.2	17.8	2.1

Country	2015 (a)	2023 (b)	Absolute change (b-a)	% of change	AAPC
Zambia	55.5	63.5	8.0	14.4	1.7
Cambodia	75.6	86.1	10.5	13.9	1.6
Morocco	55.3	60.9	5.6	10.1	1.2
Cameroon	58.8	64.6	5.8	9.9	1.2
Egypt	82.8	89.9	7.1	8.6	1.0
Indonesia	83.5	90.6	7.1	8.5	1.0
Sao Tome and Principe	83.6	89.0	5.4	6.5	0.8
Kenya	62.5	66.0	3.5	5.6	0.7
Armenia	92.8	96.0	3.2	3.5	0.4
Lesotho	74.4	76.6	2.2	3.0	0.4
Sudan	50.7	51.7	1.0	2.0	0.2
Ghana	87.3	87.8	0.5	0.6	0.1
Bhutan	84.9	84.9	0.0	0.0	0.0
Congo, Rep.	79.0	79.0	0.0	0.0	0.0
Djibouti	25.7	25.7	0.0	0.0	0.0
Guatemala	86.2	86.2	0.0	0.0	0.0
Republic of Moldova	95.4	95.4	0.0	0.0	0.0
Sri Lanka	92.5	92.5	0.0	0.0	0.0
Syrian Arab Republic	63.7	63.7	0.0	0.0	0.0
Ukraine	87.2	87.2	0.0	0.0	0.0
Vanuatu	51.8	51.8	0.0	0.0	0.0
Kyrgyz Republic	94.6	94.3	-0.3	-0.3	0.0
Honduras	88.9	88.3	-0.6	-0.7	-0.1
West Bank and Gaza	95.5	94.8	-0.7	-0.7	-0.1
Mongolia	89.6	88.5	-1.1	-1.2	-0.2
Philippines	84.3	83.0	-1.3	-1.5	-0.2
Eswatini	76.1	73.6	-2.5	-3.3	-0.4
Kiribati	70.5	67.0	-3.5	-5.0	-0.6
Solomon Islands	68.9	65.4	-3.5	-5.1	-0.7
Tunisia	85.1	79.0	-6.1	-7.2	-0.9
Papua New Guinea	54.9	49.0	-5.9	-10.8	-1.4
Myanmar	73.4	58.6	-14.8	-20.2	-2.8
Mauritania	63.0	38.5	-24.5	-38.9	-6.0
Micronesia	n/a	n/a	n/a	n/a	n/a
Samoa	n/a	n/a	n/a	n/a	n/a
Uzbekistan	n/a	n/a	n/a	n/a	n/a
Upper-middle income (n=55)					
Iraq	49.6	67.9	18.3	36.9	4.0
Turkmenistan	82.8	97.6	14.8	17.9	2.1
Albania	66.8	77.8	11.0	16.5	1.9
Russian Federation	78.3	88.0	9.7	12.4	1.5
Belize	83.1	92.6	9.5	11.4	1.4
Dominica	89.6	95.0	5.4	6.0	0.7
Ecuador	79.5	83.3	3.8	4.8	0.6
Lebanon	80.9	84.0	3.1	3.8	0.5
Algeria	67.3	69.8	2.5	3.7	0.5
Paraguay	77.4	79.6	2.2	2.8	0.4
Serbia	93.9	96.6	2.7	2.9	0.4
Guyana	92.9	95.0	2.1	2.3	0.3

Country	2015 (a)	2023 (b)	Absolute change (b-a)	% of change	AAPC
Brazil	90.9	92.9	2.0	2.2	0.3
North Macedonia	93.9	95.7	1.8	1.9	0.2
Suriname	66.8	67.5	0.7	1.1	0.1
Turkiye	88.9	89.7	0.8	0.9	0.1
Argentina	89.8	90.4	0.6	0.7	0.1
Belarus	99.7	99.9	0.2	0.2	0.0
Gabon	77.6	77.8	0.2	0.3	0.0
Azerbaijan	75.6	75.6	0.0	0.0	0.0
Bosnia and Herzegovina	84.2	84.2	0.0	0.0	0.0
Botswana	73.3	73.3	0.0	0.0	0.0
Equatorial Guinea	66.9	66.9	0.0	0.0	0.0
Iran	94.3	94.3	0.0	0.0	0.0
Jamaica	85.6	85.6	0.0	0.0	0.0
Kazakhstan	95.3	95.3	0.0	0.0	0.0
Mauritius	77.7	77.7	0.0	0.0	0.0
Namibia	62.5	62.5	0.0	0.0	0.0
Palau	81.0	81.0	0.0	0.0	0.0
Romania	76.0	76.0	0.0	0.0	0.0
Saint Lucia	90.3	90.0	-0.3	-0.3	0.0
Peru	95.6	94.7	-0.9	-0.9	-0.1
Jordan	94.5	93.1	-1.4	-1.5	-0.2
Venezuela	86.6	85.2	-1.4	-1.6	-0.2
Georgia	88.3	86.7	-1.6	-1.8	-0.2
Dominican Republic	95.4	92.6	-2.8	-2.9	-0.4
Costa Rica	97.6	94.1	-3.5	-3.6	-0.5
Maldives	85.1	81.6	-3.5	-4.1	-0.5
Saint Vincent and the Grenadines	72.9	69.8	-3.1	-4.3	-0.5
Mexico	94.3	89.6	-4.7	-5.0	-0.6
Fiji	93.6	88.7	-4.9	-5.2	-0.7
Thailand	93.4	88.3	-5.1	-5.5	-0.7
Colombia	89.9	81.6	-8.3	-9.2	-1.2
Tuvalu	67.3	60.3	-7.0	-10.4	-1.4
Panama	98.6	88.2	-10.4	-10.6	-1.4
Marshall Islands	77.1	67.8	-9.3	-12.1	-1.6
Montenegro	86.6	75.4	-11.2	-12.9	-1.7
South Africa	87.1	75.5	-11.6	-13.3	-1.8
Cuba	97.8	79.3	-18.5	-18.9	-2.6
Angola	n/a	n/a	n/a	n/a	n/a
Bulgaria	n/a	n/a	n/a	n/a	n/a
China	n/a	n/a	n/a	n/a	n/a
Grenada	n/a	n/a	n/a	n/a	n/a
Libya	n/a	n/a	n/a	n/a	n/a
Malaysia	n/a	n/a	n/a	n/a	n/a

AAPC – average annual percent change, n/a – no data available

Table S8. Country-level trends in births attended by skilled health personnel (SBA) coverage, 2015-2023

Country	2015 (a)	2023 (b)	Absolute change (b-a)	% of change	AAPC
Low income (n=31)					
Somalia	9.0	32.0	23.0	255.6	17.2
Ethiopia	16.0	50.0	34.0	212.5	15.3
South Sudan	19.0	40.0	21.0	110.5	9.8
Chad	24.0	47.0	23.0	95.8	8.8
Senegal	53.0	94.0	41.0	77.4	7.4
United Republic of Tanzania	49.0	85.0	36.0	73.5	7.1
Uganda	57.0	88.0	31.0	54.4	5.6
Togo	45.0	69.0	24.0	53.3	5.5
Mali	44.0	66.0	22.0	50.0	5.2
Gambia	57.0	84.0	27.0	47.4	5.0
Niger	35.0	51.0	16.0	45.7	4.8
Sierra Leone	60.0	87.0	27.0	45.0	4.8
Nepal	56.0	80.0	24.0	42.9	4.6
Liberia	61.0	84.0	23.0	37.7	4.1
Afghanistan	51.0	68.0	17.0	33.3	3.7
Guinea	45.0	55.0	10.0	22.2	2.5
Burkina Faso	80.0	96.0	16.0	20.0	2.3
Guinea-Bissau	45.0	54.0	9.0	20.0	2.3
Comoros	82.0	97.0	15.0	18.3	2.1
Malawi	87.0	96.0	9.0	10.3	1.2
Zimbabwe	78.0	86.0	8.0	10.3	1.2
Congo, Dem. Rep.	80.0	85.0	5.0	6.3	0.8
Madagascar	44.0	46.0	2.0	4.6	0.6
Rwanda	91.0	94.0	3.0	3.3	0.4
Benin	99.0	100.0	1.0	1.0	0.1
Central African Republic	40.0	40.0	0.0	0.0	0.0
Democratic People's Republic of Korea	100.0	100.0	0.0	0.0	0.0
Eritrea	34.0	34.0	0.0	0.0	0.0
Burundi	78.0	75.0	-3.0	-3.9	-0.5
Mozambique	73.0	68.0	-5.0	-6.9	-0.9
Haiti	49.0	42.0	-7.0	-14.3	-1.9
Lower-middle income (n=51)					
Timor-Leste	29.0	69.0	40.0	137.9	11.4
Lao PDR	40.0	80.0	40.0	100.0	9.1
Bangladesh	42.0	70.0	28.0	66.7	6.6
Kenya	62.0	89.0	27.0	43.6	4.6
Cote d'Ivoire	59.0	84.0	25.0	42.4	4.5
Yemen	45.0	61.0	16.0	35.6	3.9
Tunisia	74.0	98.0	24.0	32.4	3.6
Honduras	72.0	94.0	22.0	30.6	3.4
Zambia	63.0	80.0	17.0	27.0	3.0
Ghana	71.0	88.0	17.0	23.9	2.7
Philippines	73.0	90.0	17.0	23.3	2.7
Morocco	74.0	87.0	13.0	17.6	2.0
Pakistan	59.0	68.0	9.0	15.3	1.8
Lesotho	78.0	87.0	9.0	11.5	1.4
Bhutan	89.0	99.0	10.0	11.2	1.3

Country	2015 (a)	2023 (b)	Absolute change (b-a)	% of change	AAPC
Cambodia	89.0	99.0	10.0	11.2	1.3
Indonesia	87.0	96.0	9.0	10.3	1.2
Nicaragua	40.0	44.0	4.0	10.0	1.2
India	81.0	89.0	8.0	9.9	1.2
Samoa	83.0	89.0	6.0	7.2	0.9
Cabo Verde	91.0	97.0	6.0	6.6	0.8
Cameroon	65.0	69.0	4.0	6.2	0.8
Guatemala	66.0	70.0	4.0	6.1	0.7
Eswatini	88.0	93.0	5.0	5.7	0.7
Papua New Guinea	53.0	56.0	3.0	5.7	0.7
Egypt	92.0	97.0	5.0	5.4	0.7
Sao Tome and Principe	93.0	97.0	4.0	4.3	0.5
Viet Nam	94.0	98.0	4.0	4.3	0.5
Vanuatu	89.0	91.0	2.0	2.3	0.3
Tonga	96.0	98.0	2.0	2.1	0.3
Kyrgyz Republic	98.0	100.0	2.0	2.0	0.3
Mauritania	69.0	70.0	1.0	1.5	0.2
Mongolia	99.0	100.0	1.0	1.0	0.1
Sri Lanka	99.0	100.0	1.0	1.0	0.1
Armenia	100.0	100.0	0.0	0.0	0.0
Djibouti	87.0	87.0	0.0	0.0	0.0
Micronesia	100.0	100.0	0.0	0.0	0.0
Nigeria	100.0	100.0	0.0	0.0	0.0
Republic of Moldova	100.0	100.0	0.0	0.0	0.0
Solomon Islands	86.0	86.0	0.0	0.0	0.0
Sudan	78.0	78.0	0.0	0.0	0.0
Syrian Arab Republic	96.0	96.0	0.0	0.0	0.0
Tajikistan	98.0	98.0	0.0	0.0	0.0
Ukraine	100.0	100.0	0.0	0.0	0.0
West Bank and Gaza	100.0	100.0	0.0	0.0	0.0
Uzbekistan	100.0	99.0	-1.0	-1.0	-0.1
El Salvador	100.0	98.0	-2.0	-2.0	-0.3
Kiribati	98.0	92.0	-6.0	-6.1	-0.8
Congo, Rep.	94.0	83.0	-11.0	-11.7	-1.5
Myanmar	71.0	60.0	-11.0	-15.5	-2.1
Bolivia	88.0	72.0	-16.0	-18.2	-2.5
Upper-middle income (n=55)					
Iraq	70.0	96.0	26.0	37.1	4.0
Suriname	90.0	100.0	10.0	11.1	1.3
Costa Rica	90.0	99.0	9.0	10.0	1.2
Tuvalu	93.0	100.0	7.0	7.5	0.9
Gabon	89.0	95.0	6.0	6.7	0.8
Angola	47.0	50.0	3.0	6.4	0.8
Belize	77.0	81.0	4.0	5.2	0.6
Romania	94.0	98.0	4.0	4.3	0.5
Maldives	96.0	100.0	4.0	4.2	0.5
Peru	92.0	95.0	3.0	3.3	0.4
South Africa	94.0	97.0	3.0	3.2	0.4
Marshall Islands	90.0	92.0	2.0	2.2	0.3

Country	2015 (a)	2023 (b)	Absolute change (b-a)	% of change	AAPC
Algeria	97.0	99.0	2.0	2.1	0.3
Ecuador	94.0	96.0	2.0	2.1	0.3
Guyana	96.0	98.0	2.0	2.1	0.3
Serbia	98.0	100.0	2.0	2.0	0.3
Albania	99.0	100.0	1.0	1.0	0.1
Bulgaria	93.0	94.0	1.0	1.1	0.1
Grenada	99.0	100.0	1.0	1.0	0.1
Kazakhstan	99.0	100.0	1.0	1.0	0.1
Malaysia	99.0	100.0	1.0	1.0	0.1
Saint Lucia	99.0	100.0	1.0	1.0	0.1
Azerbaijan	100.0	100.0	0.0	0.0	0.0
Belarus	100.0	100.0	0.0	0.0	0.0
Bosnia and Herzegovina	100.0	100.0	0.0	0.0	0.0
Botswana	100.0	100.0	0.0	0.0	0.0
Brazil	98.0	98.0	0.0	0.0	0.0
China	100.0	100.0	0.0	0.0	0.0
Cuba	100.0	100.0	0.0	0.0	0.0
Dominica	100.0	100.0	0.0	0.0	0.0
Equatorial Guinea	68.0	68.0	0.0	0.0	0.0
Fiji	100.0	100.0	0.0	0.0	0.0
Georgia	100.0	100.0	0.0	0.0	0.0
Iran	99.0	99.0	0.0	0.0	0.0
Jamaica	100.0	100.0	0.0	0.0	0.0
Jordan	100.0	100.0	0.0	0.0	0.0
Lebanon	98.0	98.0	0.0	0.0	0.0
Libya	100.0	100.0	0.0	0.0	0.0
Mauritius	100.0	100.0	0.0	0.0	0.0
Montenegro	99.0	99.0	0.0	0.0	0.0
Namibia	88.0	88.0	0.0	0.0	0.0
North Macedonia	100.0	100.0	0.0	0.0	0.0
Russian Federation	100.0	100.0	0.0	0.0	0.0
Saint Vincent and the Grenadines	99.0	99.0	0.0	0.0	0.0
Thailand	100.0	100.0	0.0	0.0	0.0
Turkmenistan	100.0	100.0	0.0	0.0	0.0
Venezuela	99.0	99.0	0.0	0.0	0.0
Argentina	100.0	99.0	-1.0	-1.0	-0.1
Colombia	99.0	98.0	-1.0	-1.0	-0.1
Dominican Republic	100.0	99.0	-1.0	-1.0	-0.1
Palau	100.0	99.0	-1.0	-1.0	-0.1
Turkiye	99.0	97.0	-2.0	-2.0	-0.3
Panama	96.0	93.0	-3.0	-3.1	-0.4
Paraguay	96.0	91.0	-5.0	-5.2	-0.7
Mexico	98.0	88.0	-10.0	-10.2	-1.3

AAPC – average annual percent change

Table S9. Country-level trends in institutional delivery (FBD) coverage, 2015-2023

**Country**	**2015 (a)**	**2023 (b)**	**Absolute change (b-a)**	**% of change**	**AAPC**
Low income (n=31)					
Ethiopia	16.4	47.5	31.1	189.6	14.2
Somalia	9.4	20.7	11.3	120.2	10.4
Haiti	35.9	67.3	31.4	87.5	8.2
Sierra Leone	54.4	83.4	29.0	53.3	5.5
Nepal	55.2	79.4	24.2	43.8	4.7
Liberia	55.6	79.8	24.2	43.6	4.6
Burundi	59.5	83.9	24.4	41.0	4.4
Afghanistan	48.1	66.3	18.2	37.8	4.1
Gambia	62.6	83.7	21.1	33.7	3.7
Guinea	40.3	52.6	12.3	30.5	3.4
Uganda	57.4	73.4	16.0	27.9	3.1
Chad	21.7	27.2	5.5	25.4	2.9
Comoros	76.2	95.1	18.9	24.8	2.8
United Republic of Tanzania	50.2	62.6	12.4	24.7	2.8
Senegal	74.5	92.3	17.8	23.9	2.7
Burkina Faso	82.2	94.2	12.0	14.6	1.7
Guinea-Bissau	44.0	50.4	6.4	14.6	1.7
Central African Republic	52.5	58.3	5.8	11.1	1.3
Zimbabwe	77.0	85.5	8.5	11.0	1.3
Togo	72.5	80.0	7.5	10.3	1.2
Malawi	88.9	96.7	7.8	8.8	1.1
Benin	87.0	90.6	3.6	4.1	0.5
Mali	64.5	66.8	2.3	3.6	0.4
Rwanda	90.7	93.1	2.4	2.7	0.3
Congo, Dem. Rep.	79.9	81.5	1.6	2.0	0.3
Madagascar	37.9	38.5	0.6	1.6	0.2
Eritrea	33.6	33.6	0.0	0.0	0.0
South Sudan	11.5	11.5	0.0	0.0	0.0
Democratic People's Republic of Korea	94.7	92.2	-2.5	-2.6	-0.3
Mozambique	75.4	64.6	-10.8	-14.3	-1.9
Niger	58.8	44.6	-14.2	-24.2	-3.4
Lower-middle income (n=51)					
Timor-Leste	22.1	48.5	26.4	119.5	10.3
Lao PDR	37.5	78.2	40.7	108.5	9.6
Bangladesh	37.4	64.8	27.4	73.3	7.1
Yemen	29.8	46.9	17.1	57.4	5.8
Bhutan	63.0	94.5	31.5	50.0	5.2
Papua New Guinea	37.0	54.7	17.7	47.8	5.0
Philippines	61.1	88.4	27.3	44.7	4.7
Kenya	61.2	88.1	26.9	44.0	4.7
Cote d'Ivoire	57.4	80.9	23.5	40.9	4.4
Nigeria	35.8	49.0	13.2	36.9	4.0
Kiribati	65.9	86.1	20.2	30.7	3.4
Honduras	72.0	92.0	20.0	27.8	3.1
Pakistan	55.0	70.0	15.0	27.3	3.1
Zambia	67.4	83.8	16.4	24.3	2.8
Morocco	72.7	86.1	13.4	18.4	2.1

Country	2015 (a)	2023 (b)	Absolute change (b-a)	% of change	AAPC
Ghana	73.1	86.2	13.1	17.9	2.1
Cambodia	83.2	97.8	14.6	17.6	2.0
Lesotho	76.5	89.4	12.9	16.9	2.0
Cabo Verde	83.9	97.0	13.1	15.6	1.8
Tajikistan	76.5	88.2	11.7	15.3	1.8
Indonesia	70.4	80.4	10.0	14.2	1.7
Bolivia	82.1	93.1	11.0	13.4	1.6
India	78.7	88.6	9.9	12.6	1.5
Nicaragua	88.0	96.3	8.3	9.4	1.1
Cameroon	61.3	67.0	5.7	9.3	1.1
Egypt	86.7	94.7	8.0	9.2	1.1
Guatemala	65.0	71.0	6.0	9.2	1.1
El Salvador	91.1	99.0	7.9	8.7	1.0
Samoa	81.9	88.6	6.7	8.2	1.0
Eswatini	87.7	92.7	5.0	5.7	0.7
Sao Tome and Principe	91.0	95.4	4.4	4.8	0.6
Viet Nam	93.6	96.3	2.7	2.9	0.4
Myanmar	36.2	37.1	0.9	2.5	0.3
Sri Lanka	97.1	99.5	2.4	2.5	0.3
Kyrgyz Republic	98.3	100.0	1.7	1.7	0.2
Mongolia	98.4	99.9	1.5	1.5	0.2
Mauritania	69.3	70.0	0.7	1.0	0.1
Congo, Rep.	91.5	91.5	0.0	0.0	0.0
Djibouti	86.7	86.7	0.0	0.0	0.0
Micronesia	87.1	87.1	0.0	0.0	0.0
Republic of Moldova	98.9	98.9	0.0	0.0	0.0
Solomon Islands	84.5	84.5	0.0	0.0	0.0
Sudan	27.7	27.7	0.0	0.0	0.0
Syrian Arab Republic	78.2	78.2	0.0	0.0	0.0
Ukraine	98.9	98.9	0.0	0.0	0.0
Vanuatu	88.5	88.5	0.0	0.0	0.0
Armenia	99.4	99.3	-0.1	-0.1	0.0
Tonga	98.0	97.6	-0.4	-0.4	-0.1
Uzbekistan	99.5	99.1	-0.4	-0.4	-0.1
Tunisia	98.5	98.0	-0.5	-0.5	-0.1
West Bank and Gaza	n/a	n/a	n/a	n/a	n/a
Upper-middle income (n=55)					
Iraq	70.4	86.6	16.2	23.0	2.6
Suriname	80.0	92.9	12.9	16.1	1.9
Tuvalu	93.0	98.5	5.5	5.9	0.7
Gabon	90.2	95.1	4.9	5.4	0.7
Guyana	90.0	94.2	4.2	4.7	0.6
Dominica	96.0	99.0	3.0	3.1	0.4
Ecuador	93.7	96.3	2.6	2.8	0.3
Peru	91.0	93.3	2.3	2.5	0.3
Turkmenistan	97.8	100.0	2.2	2.3	0.3
Albania	96.7	98.6	1.9	2.0	0.2
Algeria	96.8	98.6	1.8	1.9	0.2
Paraguay	96.3	97.6	1.3	1.4	0.2

Country	2015 (a)	2023 (b)	Absolute change (b-a)	% of change	AAPC
Grenada	98.6	99.8	1.2	1.2	0.2
South Africa	94.9	95.9	1.0	1.1	0.1
Jordan	98.8	99.6	0.8	0.8	0.1
Fiji	99.0	99.7	0.7	0.7	0.1
Venezuela	94.7	95.4	0.7	0.8	0.1
Kazakhstan	99.3	99.9	0.6	0.6	0.1
Colombia	96.9	97.3	0.4	0.4	0.1
Dominican Republic	99.6	99.9	0.3	0.3	0.0
China	99.7	99.9	0.2	0.2	0.0
Botswana	99.7	99.8	0.1	0.1	0.0
Romania	99.9	99.9	0.1	0.1	0.0
Serbia	99.7	99.8	0.1	0.1	0.0
Turkiye	97.2	97.3	0.1	0.1	0.0
Azerbaijan	93.1	93.1	0.0	0.0	0.0
Bosnia and Herzegovina	99.9	99.9	0.0	0.0	0.0
Equatorial Guinea	67.3	67.3	0.0	0.0	0.0
Iran	95.3	95.3	0.0	0.0	0.0
Lebanon	96.7	96.7	0.0	0.0	0.0
Libya	100.0	100.0	0.0	0.0	0.0
Namibia	87.4	87.4	0.0	0.0	0.0
Russian Federation	98.7	98.7	0.0	0.0	0.0
Cuba	99.9	99.8	-0.1	-0.1	0.0
Saint Lucia	99.1	99.0	-0.1	-0.1	0.0
Saint Vincent and the Grenadines	98.7	98.6	-0.1	-0.1	0.0
Thailand	99.6	99.5	-0.1	-0.1	0.0
Brazil	99.1	98.9	-0.2	-0.2	0.0
Angola	45.8	45.6	-0.2	-0.4	-0.1
Belarus	100.0	99.6	-0.4	-0.4	-0.1
Costa Rica	99.3	98.9	-0.4	-0.4	-0.1
Georgia	99.8	99.4	-0.4	-0.4	-0.1
Montenegro	99.0	98.6	-0.4	-0.4	-0.1
North Macedonia	99.9	99.1	-0.8	-0.8	-0.1
Bulgaria	92.0	91.2	-0.8	-0.9	-0.1
Palau	99.6	98.7	-0.9	-0.9	-0.1
Argentina	99.5	97.8	-1.7	-1.7	-0.2
Jamaica	99.4	97.6	-1.8	-1.8	-0.2
Belize	94.2	92.1	-2.1	-2.2	-0.3
Panama	94.8	91.5	-3.3	-3.5	-0.4
Mexico	96.9	93.0	-3.9	-4.0	-0.5
Maldives	99.9	94.5	-5.4	-5.4	-0.7
Malaysia	n/a	n/a	n/a	n/a	n/a
Marshall Islands	n/a	n/a	n/a	n/a	n/a
Mauritius	n/a	n/a	n/a	n/a	n/a

AAPC – average annual percent change, n/a – no data available

Table S10. Sensitivity analyses (unweighted) of associations between maternal health-related indicators and maternal mortality ratio (MMR) in low- and middle-income countries, 2015-2023*

**Variables**	**Model 1**		**Model 2**	
	**β**	***P*-value**	**β**	***P*-value**
**Maternal health services**				
Antenatal care visits, at least four	-0.004	0.03	-0.004	0.30
Births attended by skilled health personnel	-0.01	<.001	-0.01	<.001
Institutional delivery	-0.002	0.03	-0.003	0.04
**Health system capacity**				
Density of nurses and midwives	-0.0002	0.30	-0.0002	0.28
Density of physicians	0.0001	0.68	0.0000	0.999
Out-of-pocket expenditure	-0.001	0.54	-0.002	0.19
**Socioeconomic factors**				
Current health expenditure	-0.01	0.01	-0.01	0.08
Maternal education	0.01	0.05	0.01	0.08
**Interaction**				
Maternal education x Antenatal care visits, at least four			-0.00001	0.79
**Income classification**				
Low income	ref		ref	
Lower-middle income	-0.85	<.001	-0.51	0.01
Upper-middle income	-1.45	<.001	-0.80	<.001
**Year**				
2015	ref		ref	
2016	-0.03	<.001	-0.05	<.001
2017	-0.06	<.001	-0.07	<.001
2018	-0.08	<.001	-0.08	<.001
2019	-0.09	<.001	-0.09	<.001
2020	0.01	0.79	0.01	0.80
2021	0.26	<.001	0.27	<.001
2022	-0.07	0.08	-0.06	0.14
2023	-0.17	<.001	-0.15	<.001
**Obs = 1233**		1067		1067
**N = 137**		123		123

Obs – observations, ref - reference

*Model 1 presents the fully adjusted generalized linear mixed model, including maternal health service indicators, health system capacity variables, socioeconomic factors, income classification, and year. Model 2 extends Model 1 by incorporating an interaction term between maternal education and antenatal care visits (at least 4). We specified country-level random effects to account for within-country correlation overtime.

Table S11. Associations between maternal health-related indicators and maternal mortality ratio (MMR) in low- and middle-income countries by World Bank income group and maternal education, 2015-2023

Variable	Low income						Lower-middle income						Upper-middle income					
	Low ME		Moderate ME		High ME		Low ME		Moderate ME		High ME		Low ME*		Moderate ME		High ME	
	β	*P*-value	β	*P*-value	β	*P*-value	β	*P*-value	β	*P*-value	β	*P*-value	β	*P*-value	β	*P*-value	β	*P*-value
**Maternal health services**																		
Antenatal care visits, at least four	0.002	0.53	-0.01	0.03	-0.07	0.03	0.01	0.09	-0.002	0.69	-0.01	0.17			-0.02	<.001	-0.02	0.001
Births attended by skilled health personnel	-0.001	0.82	-0.004	0.06	-0.04	0.22	0.02	0.02	-0.004	0.40	-0.002	0.55			0.03	<.001	-0.01	0.01
Institutional delivery	-0.01	0.09	-0.001	0.62	0.01	0.73	-0.02	0.03	-0.01	0.01	-0.01	0.08			-0.03	<.001	-0.04	0.02
**Health system factors**																		
Density of nurses and midwives	-0.02	0.001	-0.02	0.07	-0.01	0.02	-0.001	0.50	0.01	0.23	-0.003	0.05			-0.001	0.002	-0.01	<.001
Density of physicians	-0.06	0.07	0.03	0.30	0.01	0.11	-0.05	0.001	-0.08	0.04	0.010	0.09			0.04	<.001	-0.01	0.01
OOP expenditure	0.01	0.01	0.003	0.80	0.005	0.41	-0.002	0.75	-0.001	0.90	-0.02	0.01			-0.03	<.001	-0.02	0.01
**Socioeconomic factor**																		
Current health expenditure	0.01	0.03	0.03	0.35	-0.01	0.52	-0.002	0.96	-0.05	0.44	0.05	0.06			-0.37	<.001	0.02	0.59
**Year**																		
2015	ref		ref		ref		ref		ref		ref		ref		ref		ref	
2016	-0.05	<.001	-0.003	0.96	-0.03	0.08	-0.07	0.02	-0.04	0.49	-0.05	0.01			-0.34	0.01	-0.15	0.01
2017	-0.05	0.11	-0.01	0.92	0.02	0.79	-0.12	0.02	-0.08	0.34	-0.05	0.04			-0.29	<.001	-0.14	0.02
2018	-0.11	0.002	-0.05	0.34	-0.005	0.96	-0.16	0.001	-0.15	0.12	-0.03	0.24			-0.36	<.001	-0.15	0.04
2019	-0.11	0.09	-0.10	0.07	-0.03	0.70	-0.20	<.001	-0.13	0.20	-0.01	0.76			-0.30	0.02	-0.25	<.001
2020	-0.14	0.04	-0.19	0.09	-0.03	0.78	-0.26	0.005	-0.001	0.99	-0.01	0.86			0.16	0.03	-0.03	0.75
2021	-0.12	0.04	-0.13	0.29	0.08	0.42	0.04	0.64	0.27	0.02	0.18	0.003			-0.12	0.001	0.37	<.001
2022	-0.24	0.003	-0.21	0.07	-0.02	0.82	-0.15	0.22	0.02	0.75	-0.11	0.002			-0.70	<.001	0.07	0.63
2023	-0.30	0.001	-0.12	0.17	-0.03	0.74	-0.19	0.14	-0.09	0.21	-0.19	<.001			-0.67	<.001	-0.29	0.001
**N**		24		10		2		12		19		29		1		5		41

ME – maternal education, OOP – out-of-pocket, ref – reference

**Estimates are unavailable due to model non-convergence arising from a single observation (n = 1).*

Supplementary materials (Figures)

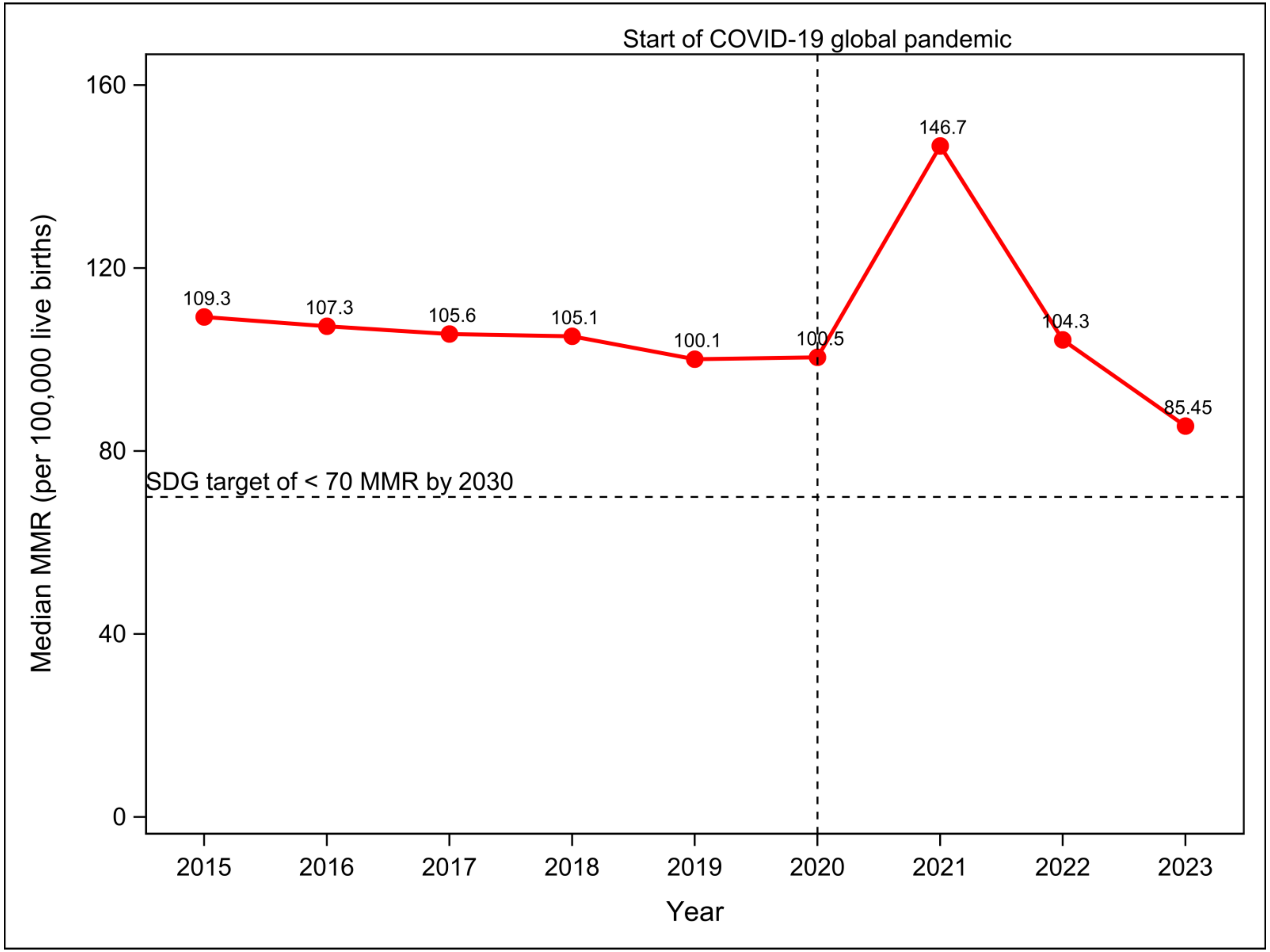

Figure S1. Trends in median maternal mortality ratio (MMR) in low- and middle-income countries (LMICs), 2015-2023. SDG – Sustainable Development Goal; COVID-19 – coronavirus disease 2019

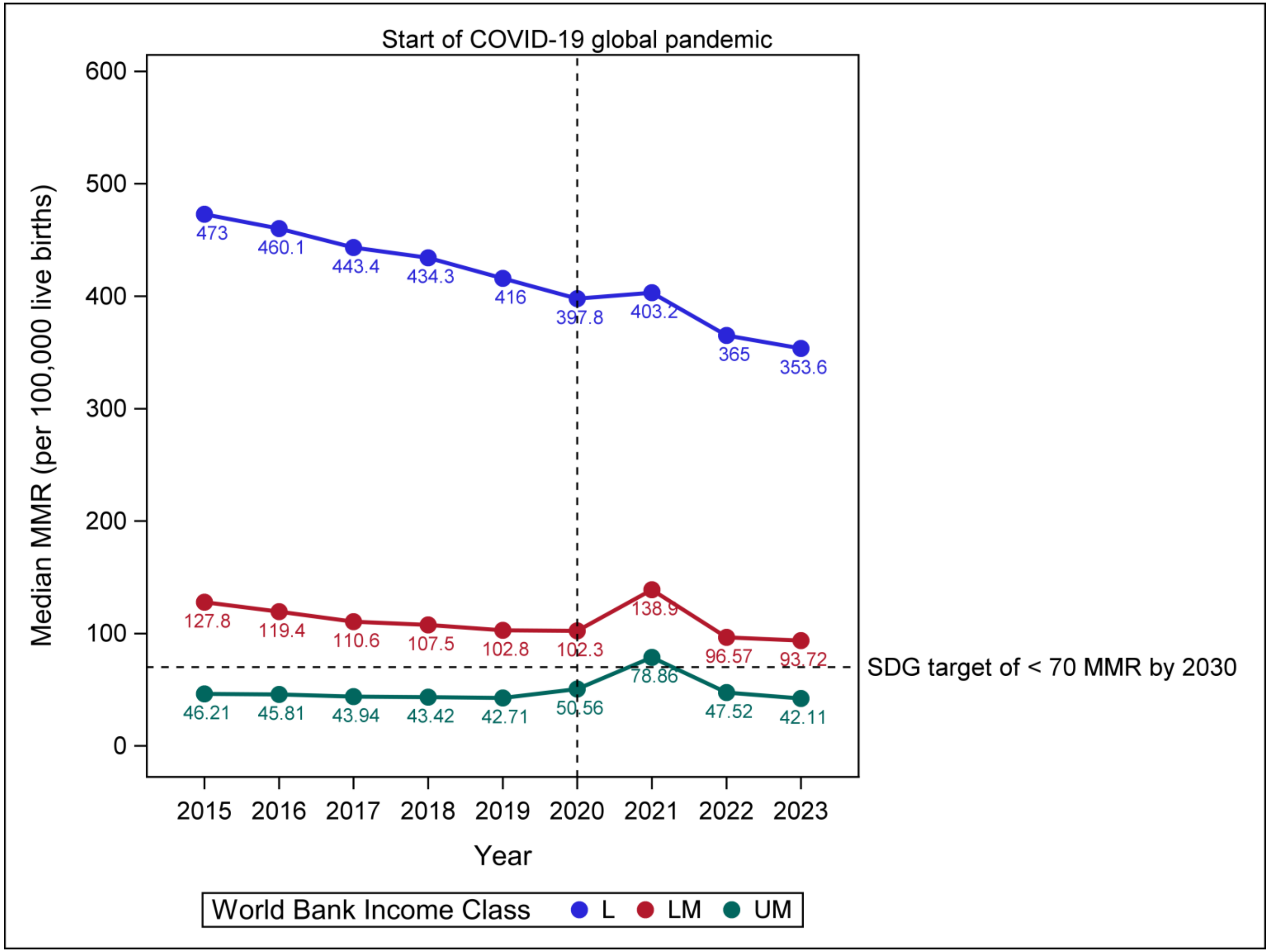

Figure S2. Trends in median maternal mortality ratio (MMR) in 137 low- and middle-income countries (LMICs) by World Bank income group, 2015-2023. L – low-income countries, LM – lower-middle-income countries, SDG – Sustainable Development Goals, UM – upper-middle-income countries

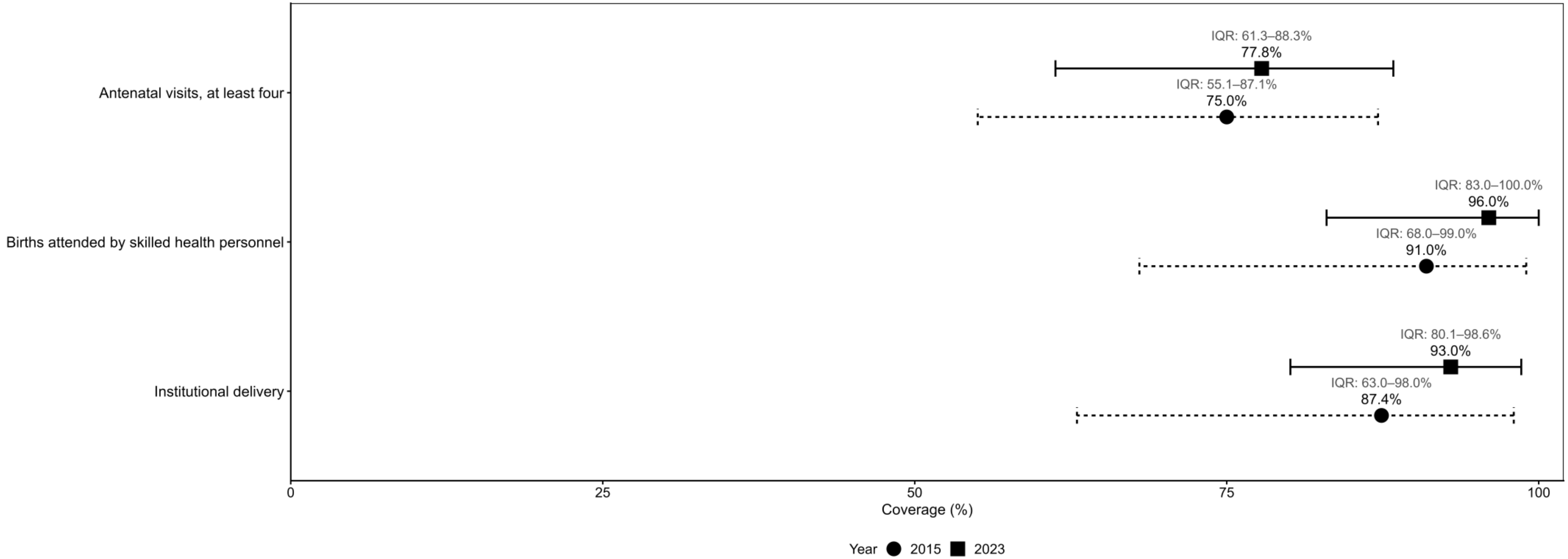

Figure S3. Overall coverage of key maternal health services (MHS) in low- and middle-income countries (LMICs). Points represent median coverage and horizontal lines indicate interquartile ranges (IQRs) across all LMICs.

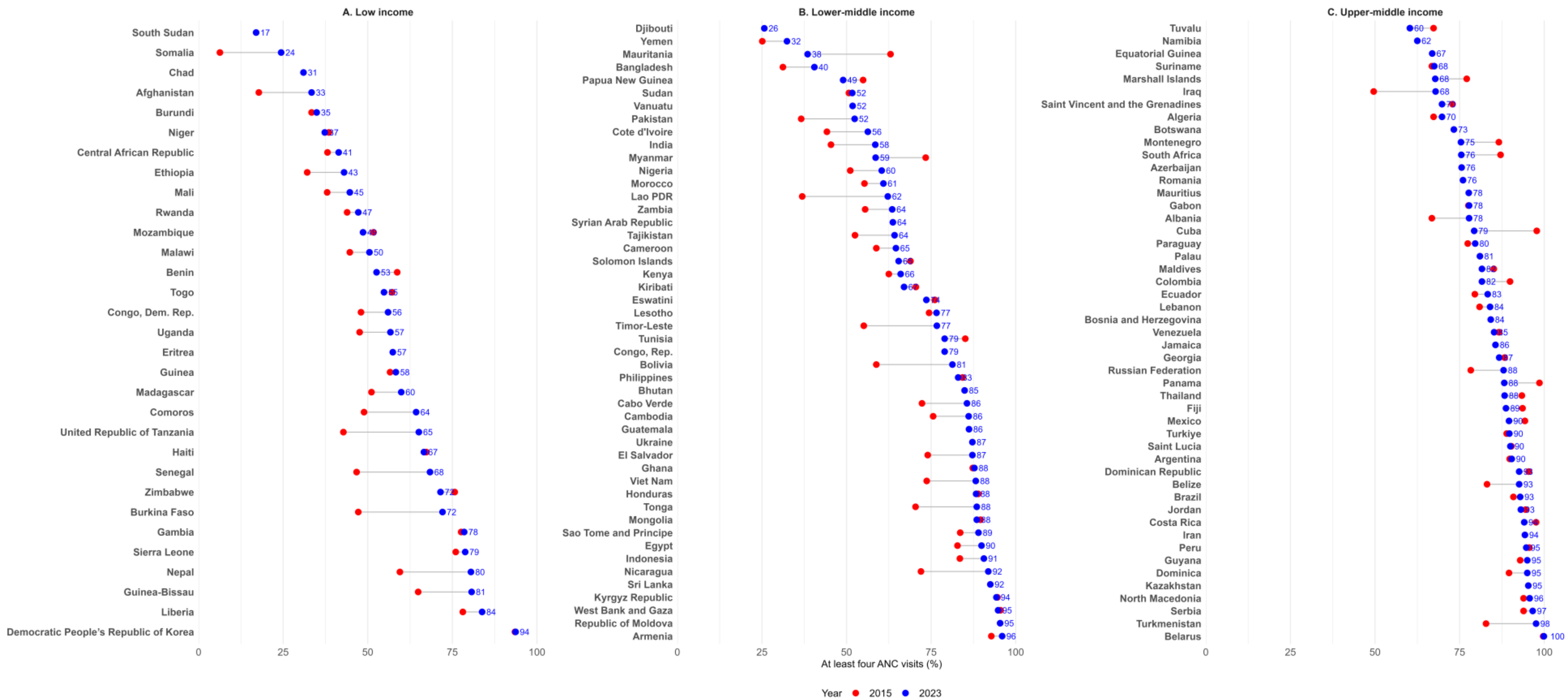

Figure S4. Country-level trends in at least four antenatal care visits (ANC4+) in low- and middle-income countries (LMICs) by World Bank income group, 2015-2023.

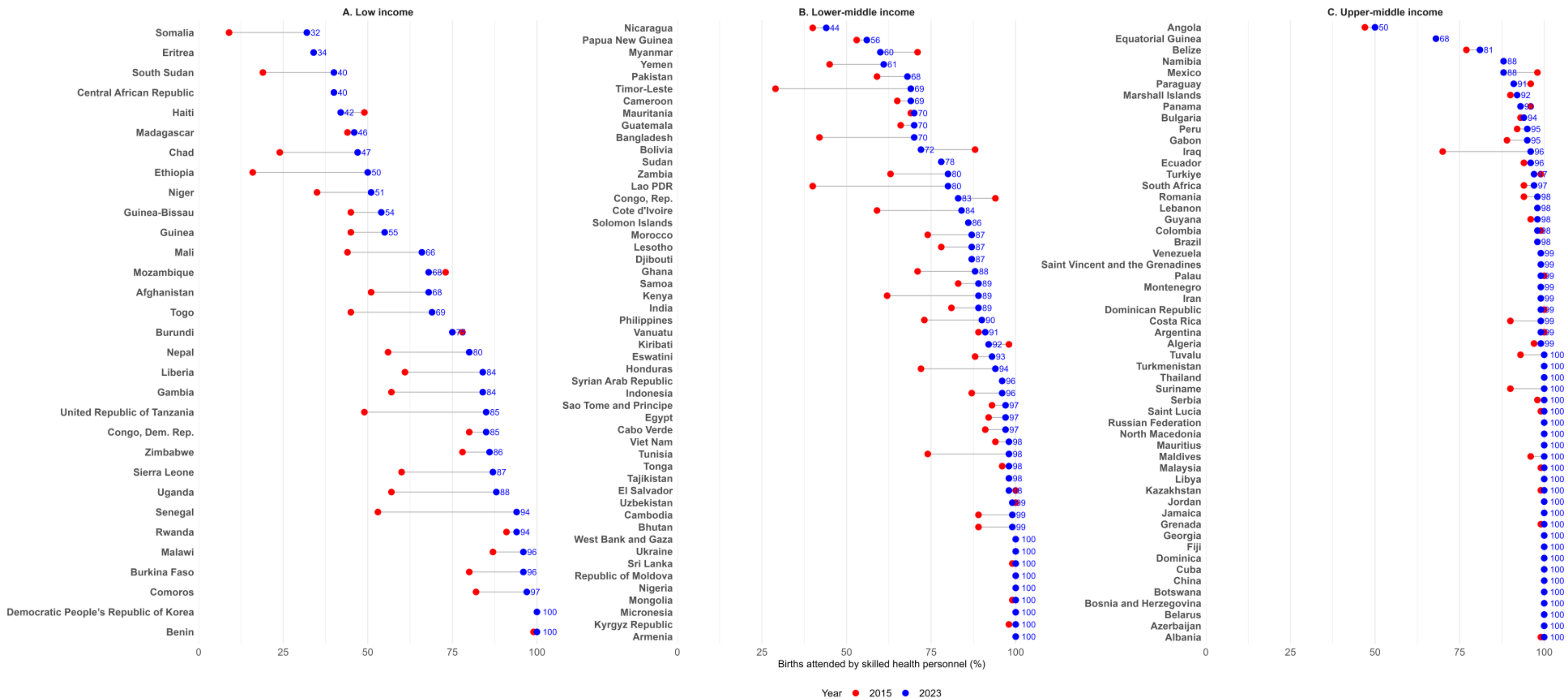

Figure S5. Country-level trends in births attended by skilled health personnel (SBA) in low- and middle-income countries (LMICs) by World Bank income group, 2015-2023

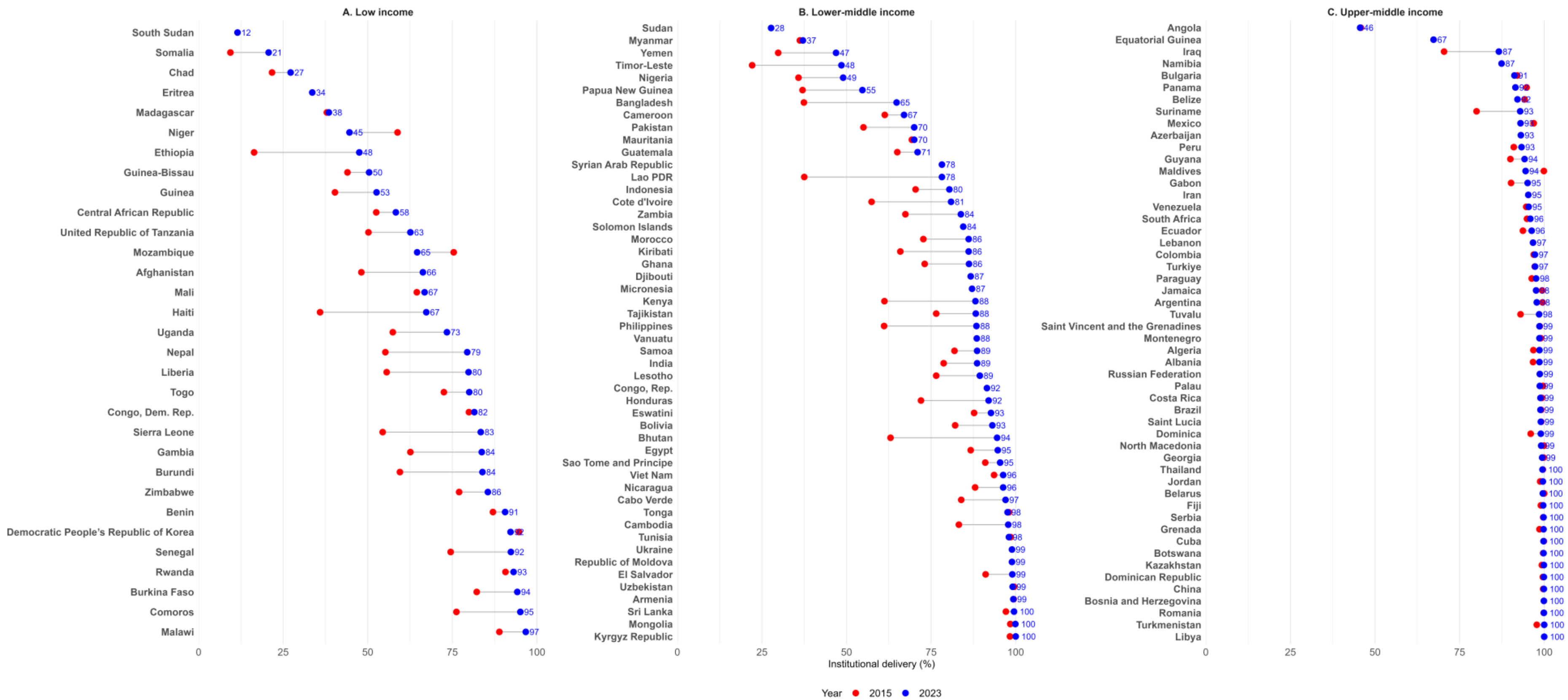

Figure S6. Country-level trends in institutional delivery (FBD) in low- and middle-income countries (LMICs) by World Bank income group, 2015-2023.

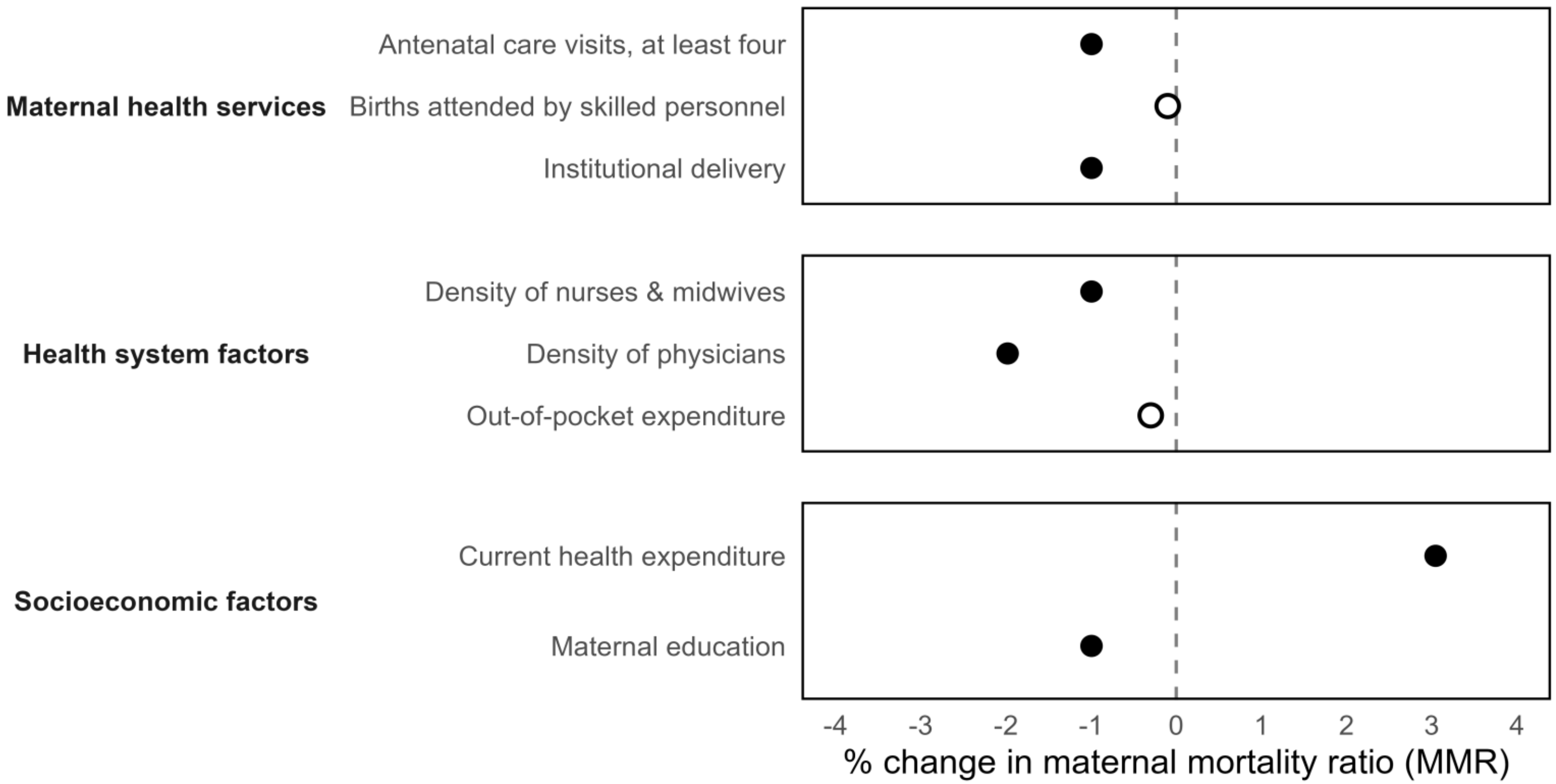

Figure S7. Multivariate-adjusted associations of maternal health services and selected health system and socioeconomic factors with maternal mortality ratio (MMR) in low- and middle-income countries (LMICs) from 2015 to 2023. Filled points indicate statistical significance ($P < 0.05$), whereas open points indicate non-significance ($P > 0.05$).

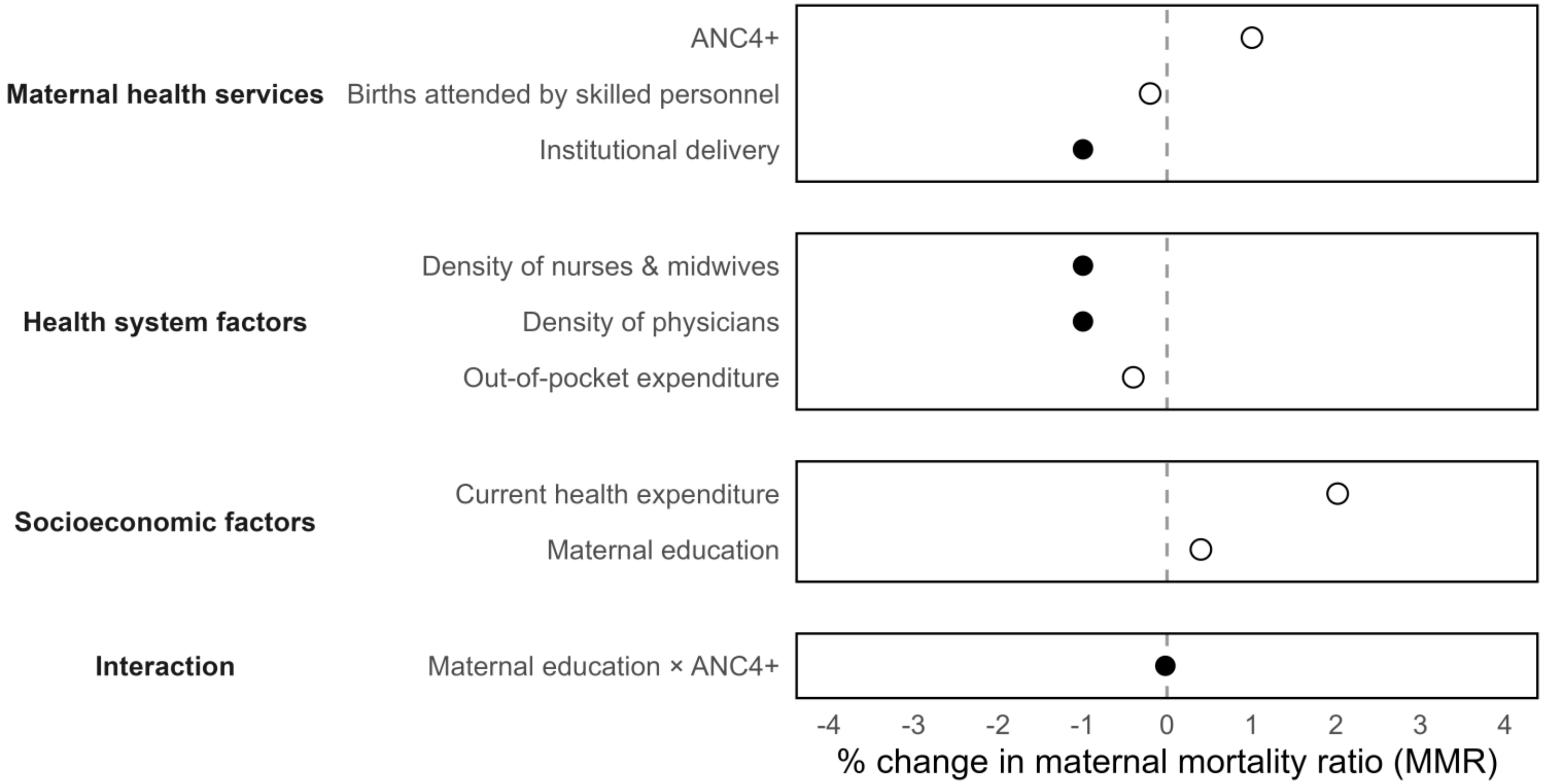

Figure S8. Multivariate-adjusted associations of maternal health services and selected health system and socioeconomic factors with maternal mortality ratio (MMR) in low- and middle-income countries (LMICs) from 2015 to 2023, including the interaction between maternal education and at least four antenatal care visits (ANC4+). Filled points indicate statistical significance ($P < 0.05$), whereas open points indicate non-significance ($P > 0.05$). ANC4+ – at least four antenatal care visits

Supplementary materials
(Files)

File S1. Adherence to JoGH's GRABDROP guideline items.

**JoGH guideline items**
1. Please list all papers published by each co-author in the previous 3 years that were based on secondary analysis of a big data repository

Francis Sarial Ganancial

1. **Ganancial, F. S.,** Cruz-Delfino, R., & Hsieh, V. C. R. (2026). Geographical disparities in maternal healthcare and mortality in the Philippines: a 10-year comparison. *International Health*, *18*(1), 83-92.

Vivian Chia-Rong Hsieh

1. Ganancial, F. S., Cruz-Delfino, R., & **Hsieh, V. C. R.** (2026). Geographical disparities in maternal healthcare and mortality in the Philippines: a 10-year comparison. *International Health*, *18*(1), 83-92.
2. Kim, S., Oh, J., Shin, J. I., Aalruz, H., Abbas, N., Abdulmalik, A. E., ... & Isa, M. A. (2026). Global burden of metabolic dysfunction-associated steatotic liver disease, 1990–2023, and projections to 2050: a systematic analysis for the Global Burden of Disease Study 2023. *The Lancet Gastroenterology & Hepatology*, *11*(6), 463-494.
3. Gray, A. P., Chung, E., Hsu, R. L., Araki, D. T., Hayoon, A. G., Weaver, N. D., ... & Fazylov, T. (2025). Global, regional, and national sepsis incidence and mortality, 1990–2021: a systematic analysis. *The Lancet Global Health*, *13*(12), e2013-e2026. doi: 10.1016/S2214-109X(25)00486-3. PMID: 41135560.
4. Force, L. M., Kocarnik, J. M., May, M. L., Bhangdia, K., Crist, A., Penberthy, L., ... & Amu, H. (2025). The global, regional, and national burden of cancer, 1990–2023, with forecasts to 2050: a systematic analysis for the Global Burden of Disease Study 2023. *The Lancet*, *406*(10512), 1565-1586.
5. **Hsieh, V. C.**, Tsai, M. H., Chiang, H. C., & Weng, M. Y. (2025). Lessons learned from Taiwan's response to the COVID-19 pandemic: Successes, challenges, and implications for future pandemics. *European Journal of Public Health, 35*(1), 153–162.
6. Yang, C. Y., Chang, C. Y. Y., Muo, C. H., **Hsieh, V. C**. R., Sung, F. C., & Chen, P. C. (2025). Risk of postpartum depression in women with allergic disorders: A nationwide cohort study involving 1,017,507 women. *Journal of Affective Disorders*, *371*, 164-169.
7. GBD 2021 Causes of Death Collaborators. (2024). Global burden of 288 causes of death and life expectancy decomposition in 204 countries and territories and 811 subnational locations, 1990–2021: a systematic analysis for the Global Burden of Disease Study 2021. *Lancet (London, England)*, *403*(10440), 2100.
8. GBD 2021 Fertility and Forecasting Collaborators. (2024). Global fertility in 204 countries and territories, 1950–2021, with forecasts to 2100: A comprehensive demographic analysis for the Global Burden of Disease Study 2021. *The Lancet, 403*(10440), 2057–2099.
9. GBD 2019 Asia and All Cancers Collaborators. (2024). Temporal patterns of cancer burden in Asia, 1990–2019: A systematic examination for the Global Burden of Disease 2019 study. *The Lancet Regional Health – Southeast Asia, 21*, Article 100333. doi: 10.1016/j.lansea.2023.100333.
10. Hsieh, C. L., Chung, C. Y., Chen, H. Y., Shieh, S. H., Hsieh, M. S., & **Hsieh, V. C. R**. (2023). Bridging geographical disparities across 368 townships with healthcare system and socioeconomic factors in Taiwan. *Scientific Reports*, *13*(1), 15007.

2. Please explain the key elements of your study design and the use of the available datasets that make your study an original scientific contribution
This study used publicly available country-level data from the WHO Global Health Observatory and World Bank databases to examine trends and disparities in maternal mortality and maternal health service coverage across 137 low- and middle-income countries from 2015 to 2023. Unlike previous studies that focused primarily on individual maternal health service indicators or single-country settings, this study integrated maternal health service, health system, and socioeconomic indicators within a longitudinal, multi-country framework. The study further assessed disparities across World Bank income groups and maternal education levels, including interaction and stratified analyses to examine whether the association between antenatal care coverage and maternal mortality varied by educational context. By combining multiple global datasets and applying population-weighted generalized linear mixed models, this study provides updated evidence on inequalities in maternal mortality and maternal health service utilization during the Sustainable Development Goals era.

3. Please list all publications that addressed similar research questions in the same dataset and indicate where you cited them in your paper
The authors have not previously published any studies addressing similar research questions using the same dataset employed in the current study. While the authors have previously published research on maternal mortality using national administrative data from the Philippines, those studies used different datasets and addressed different research objectives. Consequently, no prior publications using the same dataset were available for citation in the present manuscript.

4. Please explain how you addressed multiple testing through an appropriately rigorous statistical threshold and indicate this in the methods section
The analyses were based on a priori hypotheses about associations between maternal health service coverage and maternal mortality, with explanatory variables selected based on established theoretical and empirical evidence. Hence, a formal adjustment for multiple testing was not applied. Consistent with epidemiological practice, statistical inference was based on the magnitude and direction of effect estimates, corresponding confidence intervals (although they were not used in this current study), and p-values rather than solely on statistical significance.

5. Please declare to what extent have AI chatbots been used in developing your paper and to which parts of the paper did they contribute
In general, Artificial Intelligence (AI), including Grammarly, QuillBot, and Perplexity, was only used to edit the paper for grammatical errors and sentence flow.

File S2. Strengthening the Reporting of Observational Studies in Epidemiology (STROBE) checklist

	Item No	Recommendation	Location where the item is reported
**Title and abstract**	1	(*a*) Indicate the study's design with a commonly used term in the title or the abstract	Page 1
		(*b*) Provide in the abstract an informative and balanced summary of what was done and what was found	Page 1
**Introduction**			
Background/rationale	2	Explain the scientific background and rationale for the investigation being reported	Page 2-3
Objectives	3	State specific objectives, including any prespecified hypotheses	Page 3
**Methods**			
Study design	4	Present key elements of the study design early in the paper	Page 4
Setting	5	Describe the setting, locations, and relevant dates, including periods of recruitment, exposure, follow-up, and data collection	Page 4
Participants	6	(*a*) Give the eligibility criteria, and the sources and methods of selection of participants	Page 4
Variables	7	Clearly define all outcomes, exposures, predictors, potential confounders, and effect modifiers. Give diagnostic criteria, if applicable	Page 4-5
Data sources/ measurement	8	For each variable of interest, give sources of data and details of methods of assessment (measurement). Describe the comparability of assessment methods if there is more than one group	Page 4-5
Bias	9	Describe any efforts to address potential sources of bias	Page 5
Study size	10	Explain how the study size was arrived at	Page 4
Quantitative variables	11	Explain how quantitative variables were handled in the analyses. If applicable, describe which groupings were chosen and why	Page 5
Statistical methods	12	(*a*) Describe all statistical methods, including those used to control for confounding	Page 5
		(*b*) Describe any methods used to examine subgroups and interactions	Page 5
		(*c*) Explain how missing data were addressed	Page 5
		(*d*) If applicable, describe analytical methods taking account of the sampling strategy	Not applicable
		(*e*) Describe any sensitivity analyses	Not applicable
**Results**			
Participants	13	(a) Report numbers of individuals at each stage of study—e.g., numbers potentially eligible, examined for eligibility, confirmed eligible, included in the study, completing follow-up, and analysed	Page 6
		(b) Give reasons for non-participation at each stage	Not applicable
		(c) Consider use of a flow diagram	Not applicable
Descriptive data	14	(a) Give characteristics of study participants (eg demographic, clinical, social) and information on exposures and potential confounders	Page 6-8
		(b) Indicate number of participants with missing data for each variable of interest	Page 5 (imputation described)
Outcome data	15	Report numbers of outcome events or summary measures	Page 6-7
Main results	16	(*a*) Give unadjusted estimates and, if applicable, confounder-adjusted estimates and their precision (e.g., 95% confidence interval). Make clear which confounders were adjusted for and why they were included	Page 9

	Item No	Recommendation	Location where the item is reported
		(*b*) Report category boundaries when continuous variables were categorized	Not applicable
		(*c*) If relevant, consider translating estimates of relative risk into absolute risk for a meaningful time period	Not applicable
Other analyses	17	Report other analyses done—e.g., analyses of subgroups and interactions, and sensitivity analyses	Page 7-10
**Discussion**			
Key results	18	Summarise key results with reference to study objectives	Page 10
Limitations	19	Discuss limitations of the study, taking into account sources of potential bias or imprecision. Discuss both the direction and the magnitude of any potential bias	Page 14-16
Interpretation	20	Give a cautious overall interpretation of results, considering objectives, limitations, multiplicity of analyses, results from similar studies, and other relevant evidence	Page 10-14, 16-17
Generalisability	21	Discuss the generalisability (external validity) of the study results	Page 16-17
**Other information**			
Funding	22	Give the source of funding and the role of the funders for the present study and, if applicable, for the original study on which the present article is based	Page 18
